# Production of Diverse Beauveriolide Analogs in Closely Related Fungi: a Rare Case of Fungal Chemodiversity

**DOI:** 10.1128/mSphere.00667-20

**Published:** 2020-09-02

**Authors:** Ying Yin, Bo Chen, Shuangxiu Song, Bing Li, Xiuqing Yang, Chengshu Wang

**Affiliations:** a CAS Key Laboratory of Insect Developmental and Evolutionary Biology, CAS Center for Excellence in Molecular Plant Sciences, Shanghai Institute of Plant Physiology and Ecology, Chinese Academy of Sciences, Shanghai, China; b CAS Center for Excellence in Biotic Interactions, University of Chinese Academy of Sciences, Beijing, China; c School of Life Science and Technology, ShanghaiTech University, Shanghai, China; University of Georgia

**Keywords:** Cordycipitaceae fungi, cyclodepsipeptide, beauveriolide, chemodiversity, fungal speciation, virulence

## Abstract

Fungal chemodiversity is well known in part due to the production of diverse analogous compounds by a single biosynthetic gene cluster (BGC). Usually, similar or the same metabolites are produced by closely related fungal species under a given condition, the foundation of fungal chemotaxonomy. Here, we report a rare case of the production of the cyclodepsipeptide beauveriolides (BVDs) in three insect-pathogenic fungi. We found that the more closely related fungi Beauveria bassiana and Beauveria brongniartii produced structurally distinct analogs of BVDs, whereas the less-close relatives *B. brongniartii* and Cordyceps militaris biosynthesized structurally similar congeners under the same growth condition. It was verified that a conserved BGC containing four genes is responsible for BVD biosynthesis in three fungi, including a polyketide synthase (PKS) for the production of 3-hydroxy fatty acids (FAs) with chain length variations. In contrast to BVD production patterns, phylogenetic analysis of the BGC enzymes or enzyme domains largely resulted in the congruence relationship with fungal speciation. Feeding assays demonstrated that an FA with a chain length of eight carbon atoms was preferentially utilized, whereas an FA with a chain longer than 10 carbon atoms could not be used as a substrate for BVD biosynthesis. Insect survival assays suggested that the contribution of BVDs to fungal virulence might be associated with the susceptibility of insect species. The results of this study enrich the knowledge of fungal secondary metabolic diversity that can question the reliability of fungal chemotaxonomy.

**IMPORTANCE** Fungal chemotaxonomy is an approach to classify fungi based on the fungal production profile of metabolites, especially the secondary metabolites. We found an atypical example that could question the reliability of fungal chemical classifications in this study, i.e., the more closely related entomopathogenic species Beauveria bassiana and Beauveria brongniartii produced structurally different congeners of the cyclodepsipeptide beauveriolides, whereas the rather divergent species *B. brongniartii* and Cordyceps militaris biosynthesized similar analogs under the same growth condition. The conserved biosynthetic gene cluster (BGC) containing four genes present in each species is responsible for beauveriolide production. In contrast to the compound formation profiles, the phylogenies of biosynthetic enzymes or enzymatic domains show associations with fungal speciation. Dependent on the insect species, production of beauveriolides may contribute to fungal virulence against the susceptible insect hosts. The findings in this study augment the diversity of fungal secondary metabolisms.

## Abstract (precis)

Fungal chemotaxonomy is an approach to classify fungi based on the fungal production profile of metabolites, especially the secondary metabolites. We found an atypical example that could question the reliability of fungal chemical classifications in this study, i.e., the more closely related entomopathogenic species Beauveria bassiana and Beauveria brongniartii produced structurally different congeners of the cyclodepsipeptide beauveriolides, whereas the rather divergent species *B. brongniartii* and Cordyceps militaris biosynthesized similar analogs under the same growth condition. The conserved biosynthetic gene cluster (BGC) containing four genes present in each species is responsible for beauveriolide production. In contrast to the compound formation profiles, the phylogenies of biosynthetic enzymes or enzymatic domains show associations with fungal speciation. Dependent on the insect species, production of beauveriolides may contribute to fungal virulence against the susceptible insect hosts. The findings in this study augment the diversity of fungal secondary metabolisms.

## INTRODUCTION

It is common that different analogs of certain secondary metabolites can be produced by a fungal species or different species of related or unrelated fungi (1, 2). For example, more than 30 structural analogs of cyclosporine, the immunosuppressant drug, have been identified from the opportunistic insect-pathogenic fungus Tolypocladium inflatum and other fungi (3). For the insecticidal and phytotoxic destruxins, around 40 structural analogs with variations in bioactivities can be similarly produced by Metarhizium and other fungal species (4). It is now clear that the nonribosomal peptide synthetase (NRPS) can upload the alternate building blocks of amino acids or hydroxyl acids for cyclodepsipeptide biosynthesis (5, 6). Taken together with the function of various tailoring enzymes, diverse cyclodepsipeptide analogs can therefore be produced by a single biosynthetic gene cluster (BGC) (2). It is still rare to find that the structurally different analogs are produced by the conserved BGC in closely related fungi.

**FIG 1:**
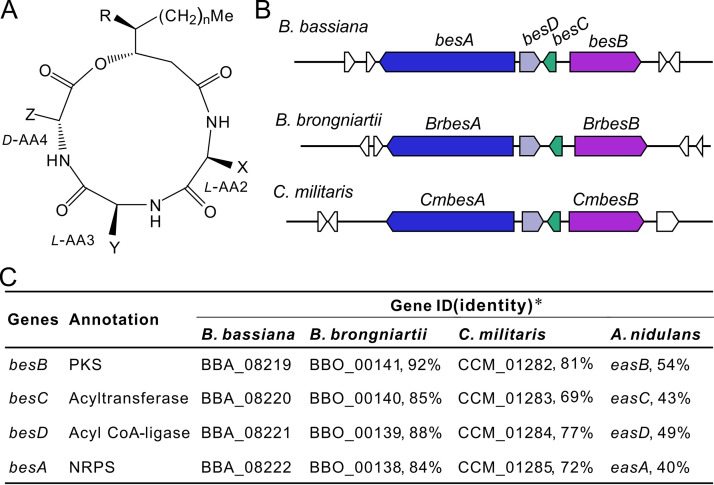
Schematic presentation of BVD structure and the biosynthetic gene cluster. (A) Common structure of BVDs. The analogs with different amino acid residues l-AA2, l-AA3, and d-AA4 as well as the letters X, Y, Z, n, and R can be referred to in Table S1 in the supplemental material. (B) Conservative relationship of the gene clusters responsible for the biosynthesis of BVDs in different fungi. The genes labeled in the same color show orthologous relationships. (C) Similarity analysis of the clustered genes in three insect pathogens. *, identity at the amino acid level compared with the homologs of B. bassiana.

The cyclodepsipeptide beauverolides were first identified in 1977 from the insect-pathogenic fungus Beauveria bassiana (7). Different congeners were later identified either from B. bassiana or from its close relatives Beauveria tenella (8), Cordyceps militaris (9), and *Paecilomyces fumosoroseus* (now classified as *Isaria fumosorosea*) (8, 10). However, the term beauveriolide instead of beauverolide was used for those analogs later identified in *C. militaris* or *Beauveria* sp. (9, 11). For simplicity, if not otherwise specified, the terms beauverolide and beauveriolide are designated BVDs that are composed of a 3-hydroxy-4-methyl fatty acid (i.e., either 3-hydroxy-4-methyldecanoic acid [HMDA; 10 carbon atoms] or 3-hydroxy-4-methyloctanoic acid [HMOA; 8 carbon atoms]) and two l-type and one d-type amino acid residues (Fig. 1A). Currently, there are 28 structurally different BVDs that have been identified (see Table S1 in the supplemental material). Given that alternate BVDs have been isolated from the closely related fungi, it is still unclear regarding the association of BVD productions with fungal speciation relationship.

10.1128/mSphere.00667-20.2 TABLE S1 Compositions of BVDs identified before and in this study. Download Table S1, PDF file, 0.5 MB. Copyright © 2020 Yin et al. 2020 Yin et al. This content is distributed under the terms of the Creative Commons Attribution 4.0 International license.

Different biological and or medicinal activities have been reported for BVDs, including antiaging (12), beta-amyloid lowering (13), and inhibition of acyl coenzyme A (acyl-CoA):cholesterol acyltransferase activity to block the synthesis of cholesteryl esters (14, 15). The analog of BVDs was detected in insect hemolymph after B. bassiana infection (16). It has also been shown that injection of wax moth (Galleria mellonella) larvae with beauverolide L could not kill insects in a dosage of less than 30 μg per larvae but induced immune responses in insects (17). It is still unclear whether BVD production is required for full fungal virulence against insect hosts.

Chemical synthesis of BVD analogs has been successful (18). However, until this study, the genetic chemistry of BVD biosynthesis remained unclear. During our ongoing studies, a recent paper reported the heterologous expression of the clustered genes from *C. militaris* to successfully detect the production of beauveriolides I and III in Aspergillus nidulans (9). We have obtained the genome information of BVD-producing fungi (19–21). In this study, BVD production and gene deletions were conducted in three Cordycipitaceae fungi of insect pathogens, including *B.* bassiana, *Beauveria brongniartii*, and *C. militaris*. It is interesting to find that the conserved BGC is responsible for the biosynthesis of BVD congeners irrespective of fungal phylogenetic associations. Substrate feeding and insect bioassays have also been performed to postulate the substrate specificity of different enzymes or domains and to determine the BVD contribution to fungal virulence against insects.

## RESULTS

### Prediction and characterization of the gene cluster.

Considering that the cyclodepsipeptide BVDs contain a 3-hydroxy fatty acid (FA) as one of the building blocks like emericellamides produced by A. nidulans (22), the putative BGC for BVD biosynthesis was deduced to contain both a polyketide synthase (PKS) and NRPS genes. Based on the reciprocal BLAST analysis with the emericellamide biosynthetic genes *easA* to *easD* (22), similar to a previous analysis (9), a conserved BGC containing four genes is present in the genomes of three entomopathogenic fungi, including B. bassiana, *B. brongniartii*, and *C. militaris* (Fig. 1B and C). The genes were termed *besA* to *besD* (for beauveriolide/beauverolide synthesis) for those in B. bassiana, which is conserved in both gene order and orientation to those of *B. brongniartii* and *C. militaris* (Fig. 1B). A reciprocal BLAST analysis of the orthologous protein identities indicated that the enzymes of B. bassiana are mostly similar to those of *B. brongniartii* (84% to 95% at the amino acid level) followed by those of *C. militaris* (69% to 81% similar) and A. nidulans (40% to 54% similar) (Fig. 1C), which is generally correlated with fungal phylogenetic relationships (23, 24).

**FIG 2:**
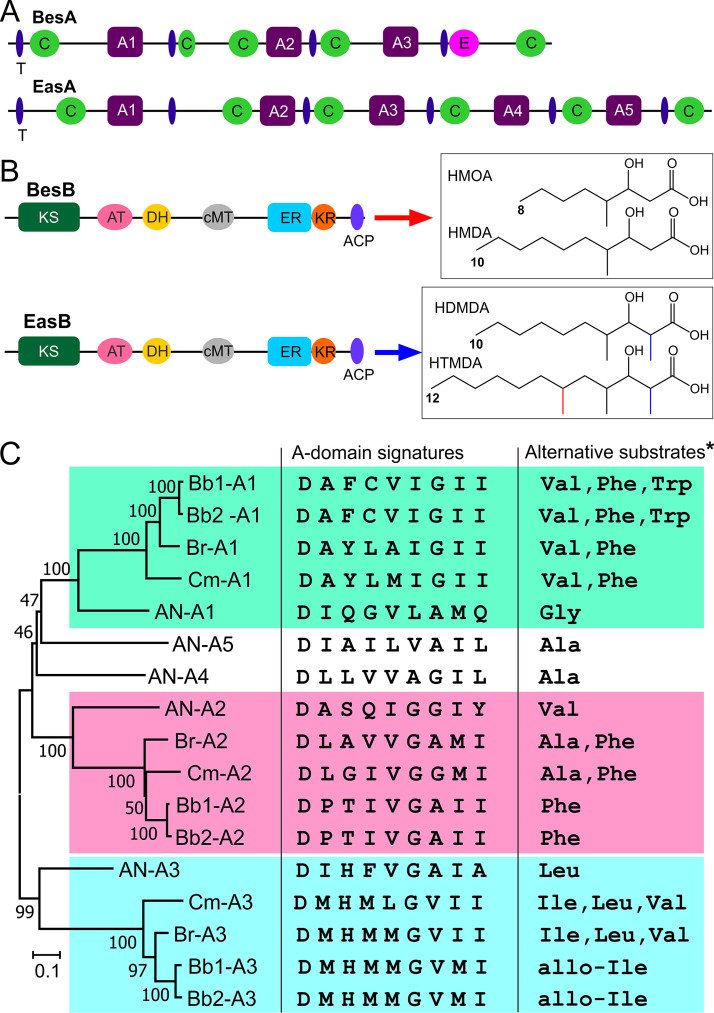
Conservation and phylogenetic analysis of the core biosynthetic enzymes. (A) Schematic structure comparison between the NRPS enzymes BesA and EasA. Different domains are as follows: C, condensation; A, adenylation; E, epimerization; T, thiolation. (B) Schematic structure comparison between the PKS enzymes BesB and EasB. Different domains are as follows: KS, ketosynthase; AT, acyl transferase; DH, dehydratase; cMT, C-methyltransferase; ER, enoyl reductase; KR, ketoreductase; ACP, acyl carrier protein. Fatty acids are as follows: HMDA, 3-hydroxy-4-methyldecanoic acid; HMOA, 3-hydroxy-4-methyloctanoic acid; HDMDA, 3-hydroxy-2,4-dimethyldecanoic acid; and HTMDA, 3-hydroxy-2,4,6-trimethyldodecanoic acid. (C) Phylogenetic, signature, and alternative substrate analysis of the NRPS A domains from different fungi. *, alternative substrates for the A1 and A2 domains are l-type amino acids, whereas the d-type amino acids are for the A3 domain. Fungal species or strains are as follows: Bb1, B. bassiana strain ARSEF 2860; Bb2, B. bassiana ARSEF 8028; Br, *B. brongniartii*; Cm, *C. militaris*; AN, A. nidulans.

Consistent with the structure difference between BVDs and emericellamides, the linear NRPS BesA is missing two modules compared with EasA. In addition, the former contains an epimerase (E) domain within the module 4 that is absent in EasA (Fig. 2A). The PKS structures are similar between BesB and EasB (54% identity); however, the former might be responsible for the biosynthesis of HMOA and HMDA, whereas EasB produces longer-chain FAs (Fig. 2B). Phylogenetic analysis of the adenylation (A) domains retrieved from homologous NRPS enzymes indicated that, except for the A2 domain, the A1 and A3 domain phylogenies were clustered by following the fungal speciation trajectory (Fig. 2C), i.e., A. nidulans diverged first and then the Cordycipitaceae fungi in the order of *C. militaris*, *B. brongniartii*, and B. bassiana (19). However, in terms of the A domain signatures and alternative substrates deduced from the BVDs produced by respective fungal species (Fig. 2C), *B. brongniartii* and *C. militaris* rather than *B. brongniartii* and *B*. bassiana might produce more similarly structured analogs.

### Verification of the BGC genes for BVD biosynthesis in different fungi.

**FIG 3:**
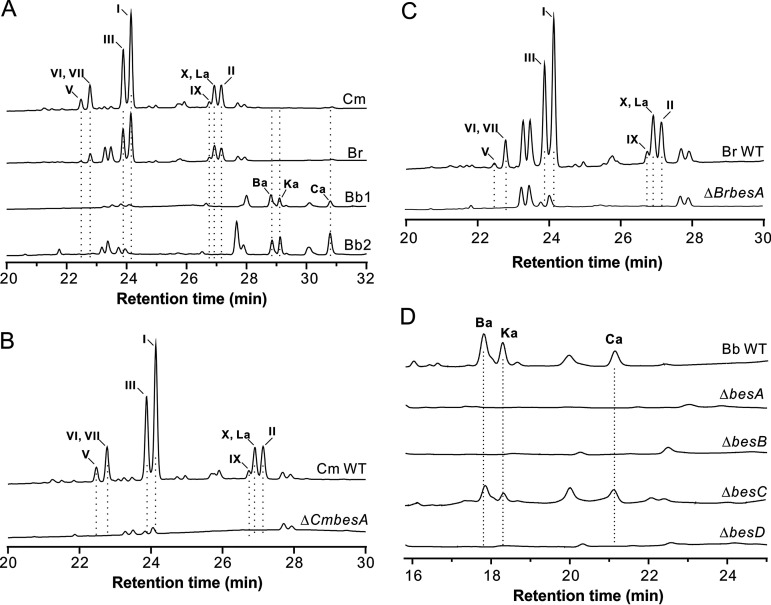
HPLC profiling of BVD production by the WT and null mutants of different fungi. (A) Production of BVD analogs by different fungal species and strains. Fungal species or strains are as shown in Fig. 2. (B) Nonproduction of different BVDs by *C. militaris* after deletion of *CmbesA*. (C) Nonproduction of different BVDs by *B. brongniartii* after deletion of *BrbesA*. (D) HPLC profiling of BVD production after deletion of the clustered genes in B. bassiana strain ARSEF 2860.

To determine BVD productions, the strains of these three fungi were incubated in a liquid culture, and the mycelia were extracted with methanol for high-performance liquid chromatography (HPLC) analysis. Intriguingly, the data revealed that the chromatographic profiles were similar between *C. militaris* and *B. brongniartii*, which were different from the similar patterns produced by two strains of B. bassiana (Fig. 3A), i.e., the more closely related B. bassiana and *B. brongniartii* produced divergent compounds.

After deletion of the core genes such as the NRPS in each species (for B. bassiana, only the strain ARSEF 2860 was used for further analysis), multiple peaks disappeared in the mutant samples (Fig. 3B to D). To determine whether these peaks are BVD analogs, the strains of B. bassiana and *B. brongniartii* were used for large-scale fermentations in Sabouraud dextrose broth (SDB) and metabolite purifications. The individually purified compounds were subject to nuclear magnetic resonance (NMR) analysis. As a result (see Text S1 in the supplemental material), 12 BVDs with alternate 3-hydroxy FAs (i.e., either HMDA or HMOA) and amino acid residues were identified in this study (see Fig. S1). A new compound was identified after two-dimensional (2D) NMR analysis (see Fig. S2; Text S1), which is composed of HMOA, l-Phe, and d-Leu and with a structure different from 28 BVDs that have been identified from different fungi (Table S1). This compound, termed beauveriolide X, was detected only from *C. militaris* and *B. brongniartii*. In summary, B. bassiana strains produced three detectable HMDA-type BVDs: Ba, Ka, and Ca. In contrast, *B. brongniartii* and *C. militaris* mostly biosynthesized HMOA-type BVDs (seven of nine, with BVDs I and III being dominant) and two HMDA-type BVDs, La and II (Table 1), a clear indication of the divergent BVD congeners produced by two *Beauveria* species.

**TABLE 1:** Structure compositions of BVD analogs identified in this study

BVD	Position 1 (FA)	Position 2 (l-AA2)	Position 3 (l-AA3)	Position 4 (d-AA4)	Producing fungus or strain
Ba	HMDA	Val	Phe	*allo*-Ile	*B.* bassiana strains ARSEF 2860 and ARSEF 8028
Ca	HMDA	Phe	Phe	*allo*-Ile	
Ka	HMDA	Trp	Phe	*allo*-Ile	
La	HMDA	Phe	Ala	*allo*-Ile	*B. brongniartii* strain RCEF 3172; *C. militaris* strain Cm01
I	HMOA	Phe	Ala	Leu	
II	HMDA	Phe	Ala	Leu	
III	HMOA	Phe	Ala	*allo*-Ile	
V	HMOA	Val	Ala	*allo*-Ile	
VI	HMOA	Val	Ala	Leu	
VII	HMOA	Phe	Ala	Val	
IX	HMOA	Phe	Phe	*allo*-Ile	
X	HMOA	Phe	Phe	Leu	

10.1128/mSphere.00667-20.1 TEXT S1 NMR data of BVDs identified in this study. Download Text S1, PDF file, 0.1 MB. Copyright © 2020 Yin et al. 2020 Yin et al. This content is distributed under the terms of the Creative Commons Attribution 4.0 International license.

10.1128/mSphere.00667-20.5 FIG S1 Structures of beauveriolides (I to X) and beauverolides (Ba, Ca, Ka, and La) identified in this study from three Cordycipitaceae fungi. HMDA, 3-hydroxy-4-methyldecanoic acid; HMOA, 3-hydroxy-4-methyloctanoic acid. Download FIG S1, TIF file, 0.8 MB. Copyright © 2020 Yin et al. 2020 Yin et al. This content is distributed under the terms of the Creative Commons Attribution 4.0 International license.

10.1128/mSphere.00667-20.6 FIG S2 1D and 2D NMR analysis of the compound beauveriolide X. NMR data (^13^C, 125 MHz; ^1^H, 500 MHz) were collected in pyridine-*d*5. HSQC, heteronuclear singular quantum correlation. HMBC, heteronuclear multiple bond correlation; COSY, correlation spectroscopy. Download FIG S2, TIF file, 0.9 MB. Copyright © 2020 Yin et al. 2020 Yin et al. This content is distributed under the terms of the Creative Commons Attribution 4.0 International license.

Contrary to that in *Beauveria* species (25), it is easy to induce sexual fruiting bodies for *C. militaris* (20). Thus, the fruiting bodies of *C. militaris* were induced both on silk moth pupae and rice medium (see Fig. S3A). In contrast to a previous report (9), metabolite extraction and HPLC analysis indicated that no detectable BVDs accumulated in either type of fruiting body samples (Fig. S3B).

10.1128/mSphere.00667-20.7 FIG S3 Fruiting body (FB) induction and metabolite profiling of *C. militaris*. (A) Phenotype of fruiting bodies formed on caterpillar pupa (left) and rice medium. The silk moth pupae were injected with spore suspensions or the rice medium were inoculated for 50 days. (B) HPLC profiling of metabolite production or nonproduction by different samples of *C. militaris*. SDB represents the extraction from the mycelial sample harvested from SDB. Download FIG S3, TIF file, 1.3 MB. Copyright © 2020 Yin et al. 2020 Yin et al. This content is distributed under the terms of the Creative Commons Attribution 4.0 International license.

### Postulation of the BVD biosynthetic pathway.

**FIG 4:**
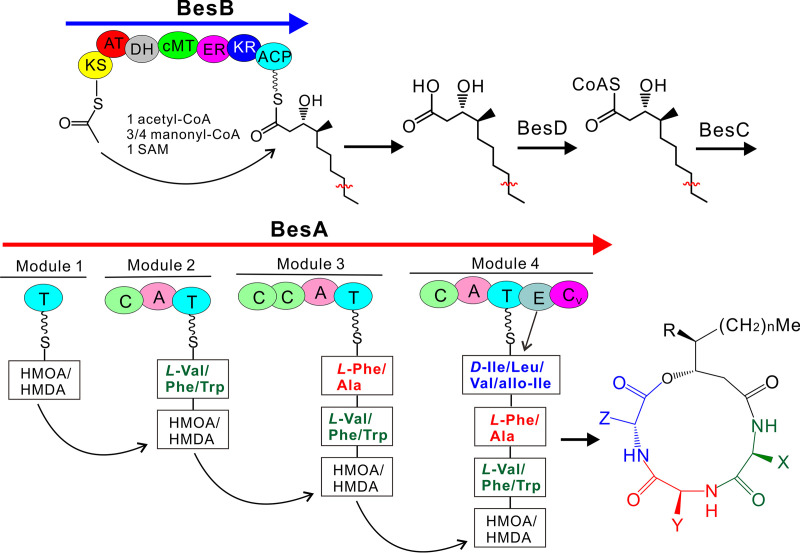
Schematic pathway for BVD biosynthesis. Different domains of the PKS enzyme are as shown in Fig. 2. Alternative substrates for the stepwise NRPS assembly line are framed for the production of BVDs detected in this study. The E domain of module 4 may be involved in epimerization of l-amino acids to d-amino acids. The analogous structures with the letters X, Y, Z, n, and R can be referred to in Table S1.

To propose the biosynthetic pathway, the putative PKS gene *besB*, acyltransferase *besC*, and CoA-ligase *besD* were also individually deleted in B. bassiana strain ARSEF 2860. Similar to that for the null mutant of the NRPS gene, Δ*besB* and Δ*besD* mutants lost their abilities to produce BVDs Ba, Ka, and Ca compared with that by the wild type (WT) strain. However, relative to the WT, a reduced amount of BVDs was still produced by the Δ*besC* mutant (Fig. 3D). Thus, the biosynthetic pathway of BVDs suggests that BesB might be responsible for the production of the 3-hydroxy FAs HMDA or HMOA by interactively catalyzing chain elongation using either three or four copies of malonyl-CoA. After the catalysis of CoA ligation by BesD, BesC might transfer CoA-HMDA or CoA-HMOA to the thiolation (T) domain of BesA for stepwise integration of alternate amino acids to form different analogs of BVDs (Fig. 4). It is noteworthy that based on the reported BVDs (Table S1), the analogous BVDs B, C, E, and F, each containing a d-Ile at position 4, previously identified in B. bassiana (26) were not detected in this study after examining two different genotypic strains.

### Substrate preference for alternate 3-hydroxy FAs and d-type amino acids.

As indicated above, B. bassiana produced the BVDs structurally different from those biosynthesized by *C. militaris* and *B. brongniartii* (Table 1; Fig. S1). We were curious about the potential mechanism(s) involved in alternative biosynthesis and integration of 3-hydroxy FAs, i.e., either HMDA or HMOA, for BVD productions in different fungi. The ketosynthase (KS), acyl transferase (AT), ketoreductase (KR) and enoyl reductase (ER) domain sequences of BesB and those of its homologs were retrieved for phylogenetic analysis. The homologous sequences of the enzymes BesC and BesD were also analyzed. It was found that, in contrast to the BVD production profiles (Fig. 3A), the obtained phylogenetic relationships of both PKS domains and catalytic enzymes were all similarly correlated with fungal speciation, i.e., the domains and enzymes of B. bassiana and *B. brongniartii* are more closely related to each other than to those of *C. militaris* (see Fig. S4).

10.1128/mSphere.00667-20.8 FIG S4 Phylogenetic analysis of the PKS domains and enzymes involved in BVD biosynthesis in different fungi. Different domains are as follows: KS, ketosynthase; AT, acyl transferase; KR, ketoreductase; ER, enoyl reductase. The domains of other PKS or other enzymes included to root the tree are as follows: OpS1 (BBA_08179), PKS for oosporein biosynthesis in B. bassiana; AfoG (AN1026), PKS for asperfuranone biosynthesis in A. nidulans; TRI101 (AAD19745) trichothecene 3-*O*-acetyltransferase of Fusarium sporotrichioides; InpC (AN3490) acyl-CoA ligase for the biosynthesis of fellutamide B in A. nidulans. Download FIG S4, TIF file, 0.4 MB. Copyright © 2020 Yin et al. 2020 Yin et al. This content is distributed under the terms of the Creative Commons Attribution 4.0 International license.

**FIG 5:**
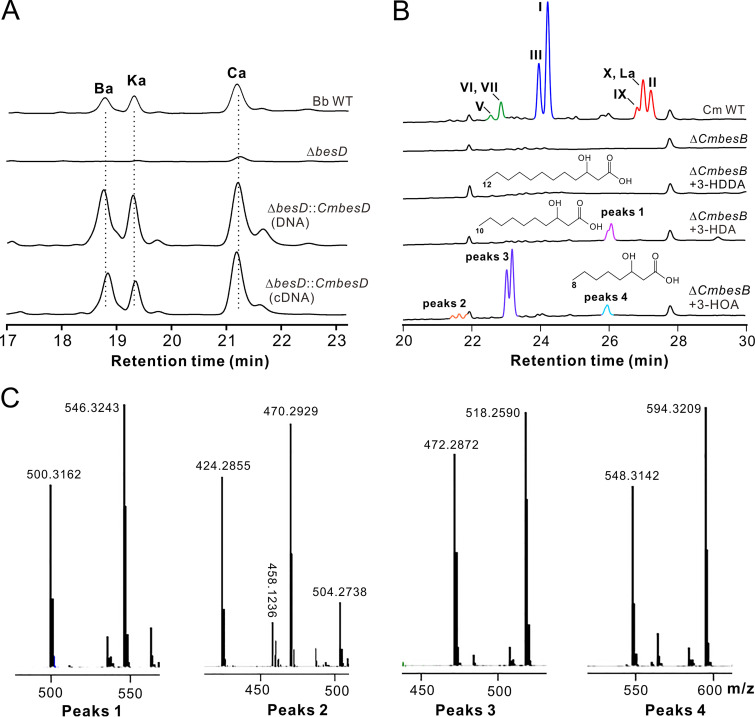
Substrate specificity tests. (A) HPLC profiling of compound production after complementation of the Δ*besD* mutant with the *CmbesD* gene. (B) HPLC profiling of metabolite production by the Δ*CmbesB* mutant after feeding with different dm-hydroxy FAs. Each FA was added at a final concentration of 100 μg/ml. FAs are as follows: 3-HDDA, 3-hydroxydodecanoic acid; 3-HAD, 3-hydroxydecanoic acid; 3-HOA, 3-hydroxyoctanoic acid. (C) Mass spectra (both [M-H]^+^ and [M+COOH]−) of the peaks detected after FA feedings of the Δ*CmbesB* mutant shown in panel B.

We next performed a gene replacement test by complementation of Δ*besD* with its orthologous gene *CmbesD* from *C. militaris*. As a result, gene rescue with either the genomic DNA or cDNA of *CmbesD* restored the ability of the Δ*besD* mutant to produce BVDs Ba, Ka, and Ca like the WT strain of B. bassiana (Fig. 5A). We also conducted feeding assays of PKS gene mutants with the available 3-hydroxy FAs, i.e., 3-hydroxydodecanoic acid (3-HDDA; 12 carbon atoms), 3-hydroxydecanoic acid (3-HDA; 10 carbon atoms), and 3-hydroxyoctanoic acid (3-HOA; 8 carbon atoms). The feeding of the Δ*besB* mutant did not produce an extra peak(s) compared with the null mutant (see Fig. S5A). However, detectable peaks were obtained after feeding the Δ*CmbesB* mutant 3-HAD and 3-HOA but not the longer chain FA 3-HDDA. It was interesting to find that more peaks were obtained after feeding with 3-HOA than with 3-HDA (Fig. 5B). The respective peaks were collected and subjected to LC-mass spectrometry (MS) analysis. Based on the obtained mass spectra of both [M-H]^+^ and [M+COOH]^−^ for each peak (Fig. 5C), 3-HDA and 3-HOA might be incorporated into the NRPS assembly line to produce demethyl (dm)-BVDs. For example, feeding with 3-HDA might lead to the formation of putative dm-BVD L and or dm-BVD La (both with a molecular weight of 501) in the Δ*CmbesB* mutant of *C. militaris*. Feeding with 3-HOA resulted in the production of at least seven dm-BVDs (see Table S2), with the putative dm-BVDs I and III (both with a molecular weight of 473) being dominant (Fig. 5B and C), which is consistent with the preference of using HMOA for BVD productions in *C. militaris* (Table 1).

10.1128/mSphere.00667-20.3 TABLE S2 Putative compositions of the compounds obtained from the mutant Δ*CmbesB* fed with 3-HDA and 3-HOA. Download Table S2, PDF file, 0.5 MB. Copyright © 2020 Yin et al. 2020 Yin et al. This content is distributed under the terms of the Creative Commons Attribution 4.0 International license.

10.1128/mSphere.00667-20.9 FIG S5 Substrate feeding assays of B. bassiana. (A) HPLC profiling of the PKS gene *besB* deletion mutant after feeding with different demethyl-hydroxy-FAs. Each FA was added at a final concentration of 100 μg/ml. 3-HDDA, 3-hydroxydodecanoic acid; 3-HAD, 3-hydroxydecanoic acid; 3-HOA, 3-hydroxyoctanoic acid. (B) HPLC profiling of B. bassiana after feeding with different concentrations of d-Val and d-Leu. The spores of the B. bassiana WT strain ARSEF 2860 were inoculated in SDB for four days, and then the d-type amino acids were added at the final concentrations as indicated for another four days. Mock control was incubated in SDB without the addition of any d-type amino acid. Download FIG S5, TIF file, 0.5 MB. Copyright © 2020 Yin et al. 2020 Yin et al. This content is distributed under the terms of the Creative Commons Attribution 4.0 International license.

The BVDs produced by B. bassiana also differ from those of *B. brongniartii* and *C. militaris* in that d-*allo*-Ile is used by B. bassiana, whereas the residues d-Leu and d-Val can be additionally integrated at position 4 by the latter fungi (Table 1). To test whether d-Leu and/or d-Val could also be uploaded by B. bassiana, feeding assays were conducted with the final concentrations of either d-type amino acid at 100 μg/ml and 500 μg/ml in SDB. As a result, no additional peak was detected after fermentations (Fig. S5B), i.e., the exogenous d-amino acids could not be directly used for BVD biosynthesis.

### Host-dependent contribution of BVDs to fungal virulence.

**FIG 6:**
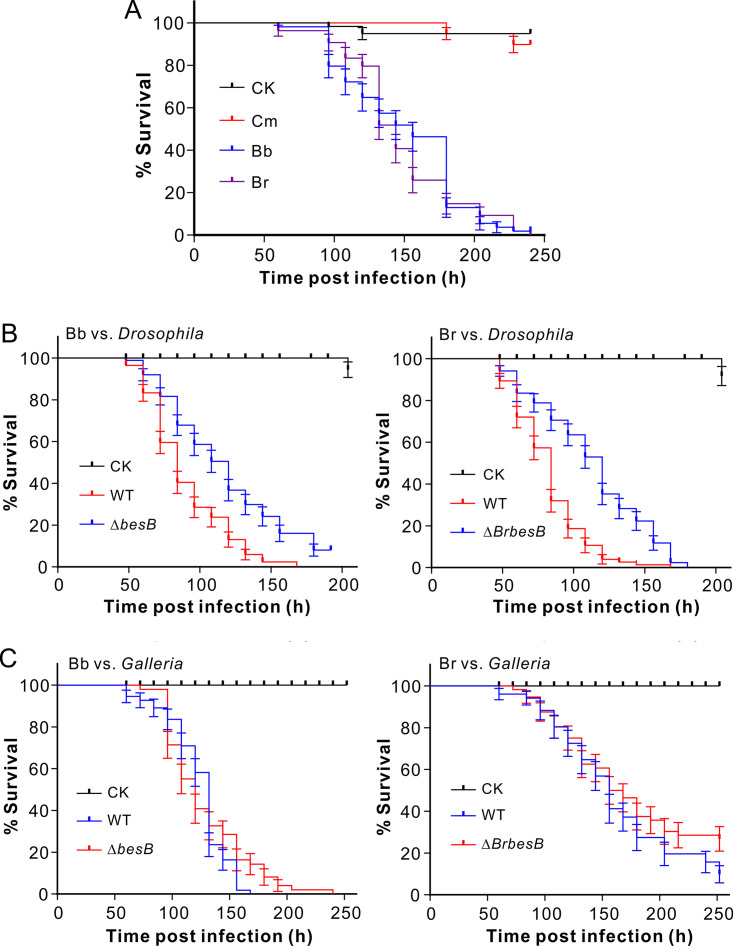
Insect survivals. (A) Survivals of fruit flies after topical infection with the spore suspensions of different WT fungi. (B) Survivals of fruit flies after topical infection with the spore suspensions of the WT and mutant fungi. (C) Survivals of the wax moth larvae after topical infection with the spore suspensions of the WT and mutant fungi. Fungal species are as follows: *C. militaris* (Cm), B. bassiana (Bb, strain ARSEF 2860), and *B. brongniartii* (Br). CK, control.

To determine whether the production of BVDs would contribute to fungal virulence, insect bioassays were first conducted with the WT strains of three fungal species. As a result, in contrast to B. bassiana (only the ARSEF 2860 strain was used) and *B. brongniartii*, *C. militaris* barely infected and killed the female adults of fruit flies (Drosophila melanogaster) (Fig. 6A). Thus, the WT and PKS gene null mutants of B. bassiana and *B. brongniartii* were used for topical infection of both fruit flies and the last instar larvae of the wax moth (Galleria mellonella). The results indicated that, relative to the corresponding WT strain, BVD-nonproducing mutants of Δ*besB* (log rank test, χ^2^ = 26.01; *P* < 0.0001) and Δ*BrbesB* (χ^2^ = 36.81; *P* < 0.0001) had significantly reduced virulence against fruit flies (Fig. 6B). However, no statistical difference (*P* > 0.05) was observed between the WT and respective mutants against the wax moth larvae (Fig. 6C). The results therefore suggested that the contribution of BVDs to fungal virulence might be dependent on the susceptibility of insect species.

## DISCUSSION

Fungal production of secondary metabolites is generally associated with fungal speciation, the typical feature and basis of fungal chemotaxonomy (27). For example, different structural destruxins are similarly produced by different toxin-producing species of Metarhizium fungi (6). The analogous mycotoxin fumonisins are selectively produced by different *Fusarium* species, such that the early diverged species (e.g., F. oxysporum and F. anthophilum) produce C-type fumonisins (with a 19-carbon atom chain), whereas the recently diverged species (e.g., F. verticillioides and F. fujikuroi) predominantly produce B-type fumonisins (with a 20-carbon atom chain) (28). We report in this study that the conserved BGC responsible for BVD biosynthesis produces structurally different analogs in three closely related Cordycipitaceae fungi. It was found that two *Beauveria* species produce distinct types of BVD analogs, whereas the more divergent species *B. brongniartii* and *C. militaris* produce similar congeners. Even the sexual stage of *Beauveria* species has been clarified to be *Cordyceps* spp. (29), different studies have shown that B. bassiana is more closely related to *B. brongniartii* than to *C. militaris* (19, 24, 30). The underlying mechanism of BGC functional divergence remains to be determined in different species. This uncommon case would at least question the reliability of fungal chemotaxonomy, especially when using a particular compound as a biomarker (31). In addition, even the similar pattern of BVD production was evident in two examined B. bassiana strains, it remains to be investigated whether different strains of the same species may produce different BVD congeners and beyond.

The evolution of fungal BGCs is still intriguing in terms of their disparate distributions in closely related or unrelated fungal species. It has been reported that BVD L and La are produced by *P. fumosorosea* (syn. *I. fumosorosea*) (8). However, our analysis of the obtained genome information revealed that the BVD BGC is missing in the genomes of *I. fumosorosea* and closely related species, such as *C. confragosa* and *C. cicadae* of Cordycipitaceae fungi (19, 30). Likewise, the conserved BGC for bibenzoquinone oosporein production is present in B. bassiana and *B. brongniartii* but absent from *C. militaris* (32). Considering that horizontal gene transfer (HGT) of BGCs frequently occurred among fungal species (1), high conservation between BVD and emericellamide BGCs would suggest the transfer of the cluster from *Aspergillus* to the ancestor of *Cordyceps* fungi followed by terminal truncation of the NRPS gene (i.e., the loss of last two modules) and BGC losses in different species such as *C. confragosa* and *C. cicadae*. Additional support of an HGT event from A. nidulans to *C. militaris* is the presence of the conserved BGC for the joint production of cordycepin and its protection molecule pentostatin (33). Nevertheless, the exact mechanism involved in the acquisition and disparate distribution of BVD BGC in different fungi remains to be determined.

The structure diversity of BVD analogs is determined via multimodular NRPS assembly of the alternate building blocks of the 3-hydroxy FAs biosynthesized by PKS and l-/d-amino acids. Even without being detected in this study, a previous report has indicated that B. bassiana strains also produce beauverolides D, E, Ea, F, and Fa that all contain a 3-hydroxy FA of HMOA (26). Taken together with the detection of BVDs Ba, Ca, and Ka (all containing a HMDA) in this study, the observations suggest that the PKS BesB might be able to produce both HMDA and HMOA. Thus, the PKS gene may not determine the specificity of alternative BVD formations in different fungi. To some extent, it is supported by our phylogenetic analysis of PKS domains (i.e., KS, AT, KR, and ER), which are all congruent with fungal speciation relationships. In addition, consistent with our feeding assays showing that a 3-hydroxy FA longer than 10 carbon atoms could not be used by *C. militaris*, heterologous expression of the BGC genes of *C. militaris* in A. nidulans only resulted in the production of BVDs I and III (9), which suggested that both 3-hydroxy-2,4-dimethyldecanoic acid (HDMDA) and 3-hydroxy-2,4,6-trimethyldodecanoic acid (HTMDA) produced by *Aspergillus* for emericellamide biosynthesis could not be integrated into the assembly line of the *Cordyceps* enzymes. We also found that the interchangeable replacement of the acyl CoA-ligase gene *besD* with *CmbesD* was not able to alter the production profile of BVDs in B. bassiana. The finding implied that both HMDA and HMOA could be similarly catalyzed to CoA-thioesters by homologous CoA-ligases and that the gene might not be involved in control of the specificity of analogous BVD productions in different fungi. We found in this study that, in contrast to the phylogenetic relationship of the NRPS A domains, A domain signature prediction would support the finding that *B. brongniartii* and *C. militaris* produce compounds with similar amino acid residues. Further experiments are still required to examine the substrate selectivity of each A domain of the NRPS enzymes.

Acyltransferase activity is responsible for the selection and integration of monomeric substrates into the megasynthases of NRPS or PKS (34). We found that the null mutant of *besC* still produced BVDs even with reduced amounts compared to that by the WT, which was in contrast to the failure of the acyltransferase mutant Δ*easC* to produce emericellamides (22). It is possible that a nonclustered acyltransferase gene (e.g., BBA_08697 with the highest [22%] identity at the amino acid level with BesC) may play a complementary role for transferring the CoA-ligated HMDA or HMOA into the NRPS assembly line; however, this remains to be investigated. Future effort is also required to replace *besC* with *CmbesC* in B. bassiana to determine whether this gene may be involved in selective uploading of CoA-thioesters and therefore the outcome of BVD diversity and specificity.

Structurally, BVDs are additionally different from emericellamides such that the former contain a d-type amino acid residue at position 4. Also, a d-alanine residue is present in the immunosuppressant agent cyclosporine, for which biosynthesis requires the function of the clustered racemase to convert l-Ala to d-Ala (5). The racemase-like gene is absent in the BGC for BVD biosynthesis. However, the difference between BesA and EasA is that the fourth module of BesA contains an epimerase (E) domain. It has been reported that the E domain of some NRPSs involves in epimerization/conversion of l-type amino acids to d-type amino acids (35, 36). Thus, this E domain may be responsible for the conversion and provision of d-amino acids from l-amino acids for BVD biosynthesis. Our feeding assays with d-Val and d-Leu did not change the BVD production profiles of B. bassiana, which would support the E domain function for *in situ* epimerization of l-amino acids to d-amino acids, i.e., either d-Val or d-Leu cannot be used as a direct substrate.

Cyclodepsipeptides produced by insect pathogenic fungi, such as beauvericin produced by B. bassiana and destruxins produced by Metarhizium species, are nonselectively insecticidal (6, 37). Our previous metabolomic analysis indicated that BVD analogs were able to be detected in insect hemolymph after the infection of B. bassiana for 36 h (16). In this study, insect bioassays indicated that the BVD-nonproducing mutants of B. bassiana and *B. brongniartii* were apparently impaired during topical infection of *Drosophila* but not in infection of the wax moth larvae compared to infection with the WT strains. The data implied that the sensitivity of different insects to BVDs might vary. As reported, injection of *Galleria* with beauveriolide L did not kill insects at a dosage of <30 μg per insect (17). The exact biological role(s) of BVDs remains to be determined in the future.

In conclusion, we report the production of the BVD congeners independent of fungal phylogenetic associations in three Cordycipitaceae fungi. The specificity of analogous BVD production is mainly determined by the A domain selectivity of the NRPS enzyme. The results of this study advance the chemodiversity of fungal secondary metabolisms that may question the reliability of fungal chemotaxonomy.

## MATERIALS AND METHODS

### Fungal strains and reagents.

The WT strains of B. bassiana ARSEF 2860 (abbreviated Bb1) and ARSEF 8028 (Bb2), *B. brongniartii* RCEF 3172 (Br), and *C. militaris* Cm01 (Cm) were used in this study for metabolite production and gene deletions. Both the WT and mutants were maintained on potato dextrose agar (PDA; BD Difco). For BVD production, the WT and mutants were inoculated in SDB (BD Difco) and incubated in a rotatory shaker at 25°C and 180 rpm for 10 days.

### Cluster prediction and genetic manipulations.

Whole-genome analysis of fungal secondary metabolic gene clusters was performed using the program antiSMASH ver. 5.0 (38). A PKS and NRPS gene cluster from three Cordycipitaceae fungi was found to be similar (40 to 60% similarity) (Fig. 1C) to the BGC of emericellamides in A. nidulans (22). To determine the functions of the clustered genes, gene deletions were individually performed by *Agrobacterium*-mediated transformation of B. bassiana (39). To generate the deletion vectors, the 5′- and 3′-flanking regions of the target gene were amplified using different primer pairs (see Table S3 in the supplemental material). For example, the primer pair BesA-U1/BesA-U2 was used to amplify the *besA* upstream region, and the primer pair BesA-L1/BesA-L2 was used to amplify the *besA* downstream region. The PCR fragments were digested with the restriction enzymes, purified, and then cloned into the same enzyme-treated binary vector pDHt-SK-Bar (for resistance against glufosinate ammonium) for transformation of the WT strain ARSEF 2860 of B. bassiana. In addition, *BrbesA* and *BrbesB* were individually deleted in *B. brongniartii*, and *CmbesA* and *CmbesB* were disrupted in *C. militaris*. The drug-resistant colonies were verified by PCR, and at least two independent null mutants of each gene were obtained by single-spore isolation for BVD production profiling. After preliminary analysis, one null mutant of each gene was used for further experiments.

10.1128/mSphere.00667-20.4 TABLE S3 PCR primers used in this study. Download Table S3, PDF file, 0.4 MB. Copyright © 2020 Yin et al. 2020 Yin et al. This content is distributed under the terms of the Creative Commons Attribution 4.0 International license.

To examine the potential association with substrate specificity, the Δ*besD* mutant of B. bassiana was complemented with the *CmbesD* gene from *C. militaris*. Thus, the full-length open reading frame of *CmbesD* was amplified using both the genomic DNA and cDNA of *C. militaris* as the templates by fusion PCRs using the ClonExpress II one-step cloning kit (Vazyme, China). The gene was under the control of the constitutive *gpdB* gene (BBA_05480) promoter (40). The obtained cassette was cloned into the plasmid pDHt-SK-Sur-GpdB (with a *sur* gene for conferring sulfonylurea resistance) (41) for transformation of the Δ*besD* mutant of B. bassiana.

### Enzyme structure modulation and phylogenetic analysis.

Modulation of the core enzymes BesA and BesB of *B.* bassiana and EasA and EasB of A. nidulans was performed using the program antiSMASH (38). The A domain signatures of NRPSs BesA and EasA were predicted with the algorithm NRPSpredictor2 (42). The A domain sequences were retrieved from the related NRPS enzymes, and the AT, ER, and KR domains were extracted from the PKS enzymes. To determine the phylogenetic relationship in association with substrate specificity, the domain sequences were aligned with the program MUSCLE (43). and the neighbor-joining trees were generated using the software MEGA X with a Dayhoff model and 1,000 bootstrap replicates (44). In addition, the homologs of BesC and BesD were also retrieved from different fungi for phylogenetic analysis.

### BVD production and chromatography analysis.

The conidia of the WT and different mutants of B. bassiana, *B. brongniartii*, and *C. militaris* were harvested from 2-week-old PDA plates and suspended in 0.05% Tween 20 to a final concentration of 1 × 10^8^ spores/ml. The spore suspensions were inoculated (1 ml each) in SDB medium (100 ml in each 250-ml flask) and incubated at 25°C and 180 rpm in a rotatory shaker for 10 days. The mycelia of each sample were then harvested by filtration, washed twice with sterile water, and extracted twice with 50 ml methanol for 30 min under sonication. The extracted samples were concentrated under vacuum, and the crude extract was dissolved in 1.5 ml methanol. There were three replicates for each strain.

Considering that the fruiting bodies of *C. militaris* are mainly consumed as health-beneficial mushrooms (45), the fruiting bodies of this fungus were also induced both on Chinese Tussah silk moth (Antheraea pernyi) pupae and on rice medium (46). After inoculation for 50 days, the fruiting bodies were harvested and freeze-dried for methanol extraction. All samples were centrifuged at 10,000 × *g* for 2 min, and the supernatants were further filtered through a 0.45-μm-pore-sized filter prior to HPLC analysis using an LC-20AD system (Shimadzu, Japan) equipped with an SPD-20A UV-visible (UV-Vis) detector and a C_18_ reverse-phase column (particle size, 5 μm; 4.6 mm by 250 mm; CNW Athena C_18_, China). Sample aliquots (15 μl each) were eluted with deionized water (solution A) and acetonitrile (solution B, 0 to 20 min, 15% to 80% acetonitrile; 20 to 35 min, 80% acetonitrile) at a flow rate of 1 ml/min and monitored at 194 nm. The column temperature was set at 40°C.

### Compound purification and structure analysis.

For purification of BVDs from B. bassiana, mycelia were harvested from 12 liters of SDB broth and extracted with methanol to generate approximately 10 g of crude extract. The crude sample was first fractionated using an Inertsil ODS (octadecyl silica) column with a gradient elution of deionized water (solution A) and methanol (solution B, 30% to 100% methanol). BVDs were concentrated in the 80% methanol fraction, which was further purified using an LC-20AD HPLC system with a C_18_ reverse-phase column (particle size, 5 μm; 10 mm by 250 mm; CNW Athena C_18_, China). Samples were eluted with deionized water (solution A) and acetonitrile (solution B, 80% acetonitrile) at a flow rate of 4 ml/min.

For purification of BVDs produced by *B. brongniartii*, mycelia were collected from 2 liters of SDB culture broth and extracted with methanol to generate crude extract for purification with an LC-20AD HPLC system. Three fractions enriched with BVDs were collected. Beauveriolides V, VI, and VII were concentrated in fraction 1. Beauveriolides I and III were concentrated in fraction 2. Beauveriolides II, IX, and X and beauverolide La were concentrated in fraction 3. These three fractions were further purified with the LC-20AD HPLC system to obtain individual BVDs. The purified compounds were individually subject to one-dimensional (1D) NMR analysis in pyridine-*d*5 to collect the ^1^H and ^13^C spectra using a Bruker Avance III-500 system equipped with a 5-mm PABBO BB-^1^H/D probe. Standard pulse parameters were used for all NMR experiments. For the new compound beauveriolide X, 2D NMR spectra were also collected to obtain the data of heteronuclear singular quantum correlation (HSQC), heteronuclear multiple bond correlation (HMBC), and correlation spectroscopy (COSY). All spectrum data were processed using the inbuilt program MestReNova (ver. 9.0.1; Metrelab Research, Santiago de Compostela, Spain).

### Substrate feeding assays.

To determine the substrate specificity, three 3-hydroxy FAs with chain length variations, i.e., 3-HDDA, 3-HAD, and 3-HAD, were ordered (Abcam, Cambridge, UK) and used to feed both the Δ*besB* mutant of B. bassiana and the Δ*CmbesB* mutant of *C. militaris*. The cultures were grown in SDB supplemented with each FA at a final concentration of 100 μg/ml at 5 days postinoculation and kept for cultivation for an additional 5 days. After incubation, mycelial samples were harvested for metabolite extraction with methanol as described above. Relative to the Δ*CmbesB* sample, additional peaks obtained in individual FA feeding experiments were collected and subjected to LC-MS analysis. Compound structures were deduced based on the obtained mass data. To determine the effect of d-type amino acid additions on BVD production, d-Leu and d-Val were used to feed the WT strain ARSEF 2860 of B. bassiana. The spores were inoculated in 250-ml flasks containing 100 ml SDB for 4 days, and d-Leu and d-Val were individually added at the final concentrations of 100 μg/ml and 500 μg/ml, respectively. The samples were incubated for another 4 days, and mycelia were harvested and extracted with methanol for HPLC analysis.

### Insect bioassays.

To determine if there was any contribution of BVDs to fungal virulence against insects, the WT strains of B. bassiana (ARSEF 2860), *B. brongniartii*, and *C. militaris* were first examined using the female adults of Drosophila melanogaster by topical infection with spore suspensions (1 × 10^7^ conidia/ml) (39). After verification that *C. militaris* was nonvirulent to fruit flies, the WT strains of B. bassiana and *B. brongniartii* and *besB* and *BrbesB* deletion mutants were then assayed in parallel against the fruit flies and the last instar larvae of the wax moth G. mellonella as described before (40). Insect mortality was recorded every 12 h, and the survival dynamics were compared between the WT and mutant of each species by Kaplan-Meier analysis (47).
