# Supplementary material for: Production of Diverse Beauveriolide Analogs in Closely Related Fungi: a Rare Case of Fungal Chemodiversity
Source: mSphere. 2020 Sep 2;5(5):e00667-20. doi: 10.1128/mSphere.00667-20 (PMC7471007; doi:10.1128/mSphere.00667-20)
Supplement: TABLE S1 [file mSphere.00667-20-st001.pdf]

Table S1 Composition of BVDs identified before and in this study.

	Compound	n	R	*L*-AA2	*L*-AA3	*D*-AA4	MW	Ref.
Beauverolide	A	5	Me	Phe	Val	Val	529	(1)
	B	5	Me	Val	Phe	Ile	543	(1)
	Ba*	5	Me	Val	Phe	allo-Ile	543	(1, 2)
	C	5	Me	Phe	Phe	Ile	591	(1)
	Ca*	5	Me	Phe	Phe	allo-Ile	591	(1)
	D	3	Me	Phe	Val	Val	501	(1)
	E	3	Me	Val	Phe	Ile	515	(1)
	Ea	3	Me	Val	Phe	allo-Ile	515	(1)
	F	3	Me	Phe	Phe	Ile	563	(1)
	Fa#**	3	Me	Phe	Phe	allo-Ile	563	(1)
	H	4	H	Phe	Ala	Leu	487	(3)
	I	6	H	Phe	Ala	Leu	515	(3)
	Ja	3	Me	Trp	Phe	allo-Ile	602	(4)
	Ka*	5	Me	Trp	Phe	allo-Ile	630	(4)
	L#**	5	Me	Phe	Ala	Leu	515	(5)
	La**	5	Me	Phe	Ala	allo-Ile	515	(5)
	M#**	3	Me	Val	Ala	Leu	439	(6)
	N	3	Me	Try	Ala	Leu	503	(6)
	P	5	Me	Val	Ala	Leu/allo-Ile	467	(6)
	Q	3	Me	Trp	Ala	Leu/allo-Ile	526	(7)
	R	5	Me	Trp	Ala	Leu/allo-Ile	554	(7)
	S	3	Me	Ala	Ala	Leu/allo-Ile	411	(8)
Beauveriolide	I**	3	Me	Phe	Ala	Leu	487	(9)
	II#**	5	Me	Phe	Ala	Leu	515	(9)
	III**	3	Me	Phe	Ala	allo-Ile	487	(10)
	IV	3	Me	Val	Ala	Val	425	(11)
	V**	3	Me	Val	Ala	allo-Ile	439	(11)
	VI#**	3	Me	Val	Ala	Leu	439	(11)
	VII**	3	Me	Phe	Ala	Val	473	(11)
	VIII	5	Me	Val	Ala	allo-Ile	467	(11)
	IX#**	3	Me	Phe	Phe	allo-Ile	563	(11)
	X**	3	Me	Phe	Phe	Leu	563	This study

Note: #, Beauverolide Fa is structurally same to Beauveriolide IX, so as beauverolide L vs. beauveriolide II, and beauverolide M vs. beauveriolide VI. *: Beauverolide Ba/Ca/Ka were identified from *B. bassiana* strains ARSEF 2860 and ARSEF 8028. **: Beauveriolide I-III/V-VII/IX-X and beauverolide La were isolated from *B. brongniartii* (Br) and *C. militaris* (Cm), among which beauveriolide X is a new compound identified in this study.

References

1. Elsworth JF, Grove JF. 1980. Cyclodepsipeptides from *Beauveria bassiana*. Part 2. Beauverolides A to F and their relationship to isarolide. J Chem Soc, Perkin Trans 1 1:1795-1799.
2. Isogai A, Kanaoka M, Matsuda H, Hori Y, Suzuki A. 1978. Structure of a new cyclodepsipeptide, beauverilide A from *Beauveria bassiana*. Agricultural and Biological Chemistry 42:1797-1798.

3. Elsworth JF, Grove JF. 1977. Cyclodepsipeptides from *Beauveria bassiana* Bals. Part 1. Beauverolides H and I. J Chem Soc, Perkin Trans 1:270-273.
4. Grove JF. 1980. Cyclodepsipeptides from *Beauveria bassiana*. Part 3. The isolation of beauverolides Ba, Ca, Ja, and Ka. J Chem Soc, Perkin Trans 1:2878-2880.
5. Jegorov A, Sedmera P, Matha V, Simek P, Zahradnickova H, Landa Z, Eyal J. 1994. Beauverolides L and La from *Beauveria tenella* and *Paecilomyces fumosoroseus*. Phytochemistry 37:1301-1303.
6. Kuzma M, Jegorov A, Kacer P, Havlicek V. 2001. Sequencing of new beauverolides by high-performance liquid chromatography and mass spectrometry. J Mass Spectrom 36:1108-1115.
7. Jegorov A, Paizs B, Kuzma M, Zabka M, Landa Z, Sulc M, Barrow MP, Havlicek V. 2004. Extraribosomal cyclic tetradepsipeptides beauverolides: profiling and modeling the fragmentation pathways. J Mass Spectrom 39:949-960.
8. Jegorov A, Hajduch M, Sulc M, Havlicek V. 2006. Nonribosomal cyclic peptides: specific markers of fungal infections. J Mass Spectrom 41:563-576.
9. Mochizuki K, Ohmori K, Tamura H, Shizuri Y, Nishiyama S, Miyoshi E, Yamamura S. 1993. The structures of bioactive cyclodepsipeptides, beauveriolides I and II, metabolites of entomopathogenic fungi *Beauveria* sp. Bull Chem Soc Jpn 66:3041-3046.
10. Namatame I, Tomoda H, Tabata N, Si S, Omura S. 1999. Structure elucidation of fungal beauveriolide III, a novel inhibitor of lipid droplet formation in mouse macrophages. J Antibiot (Tokyo) 52:7-12.
11. Matsuda D, Namatame I, Tomoda H, Kobayashi S, Zocher R, Kleinkauf H, Omura S. 2004. New beauveriolides produced by amino acid-supplemented fermentation of *Beauveria* sp. FO-6979. J Antibiot (Tokyo) 57:1-9.
