# Supplementary material for: Production of Diverse Beauveriolide Analogs in Closely Related Fungi: a Rare Case of Fungal Chemodiversity
Source: mSphere. 2020 Sep 2;5(5):e00667-20. doi: 10.1128/mSphere.00667-20 (PMC7471007; doi:10.1128/mSphere.00667-20)
Supplement: TEXT S1 [file mSphere.00667-20-s0001.pdf]

Text S1. NMR data of BVDs identified in this study.

Beauveriolide I: ^{1}H-NMR (500 MHz, pyridine-*d*5): *δ* 10.01 (1H, d, *J* = 7.4 Hz, L-Phe-2-NH), 9.82 (1H, d, *J* = 7.2 Hz, L-Ala-2-NH), 7.55 (1H, s, D-Leu-2-NH), 7.23-7.26 (5H, m, L-Phe-H-5, 6, 7, 8, 9), 5.20-5.25 (2H, m, HMOA-H-3, D-Leu-H-2), 4.83 (1H, q, *J* = 8.0 Hz, L-Phe-H-2), 4.25 (1H, m, L-Ala-H-2), 3.34 (1H, dd, *J* = 13.3, 8.5 Hz, L-Phe-H-3a), 3.20 (1H, dd, *J* = 13.4, 7.5 Hz, L-Phe-H-3b), 2.78 (1H, dd, *J* = 13.5, 4.3 Hz, HMOA-H-2a), 2.70 (1H, dd, *J* = 13.4, 10.9 Hz, HMOA-H-2b), 2.09 (1H, m, HMOA-H-4), 1.69 (3H, d, *J* = 6.9 Hz, L-Ala-H-3), 1.61-1.71 (3H, m, D-Leu-H-3, 4), 1.44 (1H, m, HMOA-H-5a), 1.16-1.22 (4H, m, HMOA-H-6, 7), 0.96 (1H, m, HMOA-H-5b), 0.90 (3H, d, *J* = 6.0 Hz, D-Leu-H-5), 0.83 (3H, d, *J* = 6.0 Hz, D-Leu-H-6), 0.78 (3H, t, *J* = 7.0 Hz, HMOA-H-8), 0.69 (3H, d, *J* = 6.9 Hz, HMOA-4-CH_3). ^{13}C-NMR (125 MHz, pyridine-*d*5): *δ* 171.8 (L-Phe-C-1), 171.6 (HMOA-C-1), 171.5 (L-Ala-C-1), 170.0 (D-Leu-C-1), 138.0 (L-Phe-C-4), 129.5 (L-Phe-C-5, 9), 128.5 (L-Phe-C-6, 8), 126.7 (L-Phe-C-7), 76.1 (HMOA-C-3), 57.5 (L-Phe-C-2), 53.0 (D-Leu-C-2), 50.1 (L-Ala-C-2), 41.6 (D-Leu-C-3), 36.6 (HMOA-C-2), 36.2 (L-Phe-C-3), 36.0 (HMOA-C-4), 30.7 (HMOA-C-5), 29.5 (HMOA-C-6), 25.0 (D-Leu-C-4), 22.9 (HMOA-C-7), 22.2 (D-Leu-C-5), 22.1 (D-Leu-C-6), 15.7 (L-Ala-C-3), 15.3 (HMOA-4-CH_3), 13.9 (HMOA-C-8).

Beauveriolide II: ^{1}H-NMR (500 MHz, pyridine-*d*5): *δ* 10.01 (1H, d, *J* = 7.3 Hz, L-Phe-2-NH), 9.82 (1H, d, *J* = 7.1 Hz, L-Ala-2-NH), 7.56 (1H, s, D-Leu-2-NH), 7.21-7.26 (5H, m, L-Phe-H-5, 6, 7, 8, 9), 5.21-5.27 (2H, m, HMDA-H-3, D-Leu-H-2), 4.83 (1H, q, *J* = 7.8 Hz, L-Phe-H-2), 4.24 (1H, m, L-Ala-H-2), 3.34 (1H, dd, *J* = 13.4, 8.6 Hz, L-Phe-H-3a), 3.20 (1H, dd, *J* = 13.3, 7.5 Hz, L-Phe-H-3b), 2.79 (1H, dd, *J* = 13.4, 4.2 Hz, HMDA-H-2a), 2.72 (1H, dd, *J* = 13.4, 10.9 Hz, HMDA-H-2b), 2.08 (1H, m, HMDA-H-4), 1.69 (3H, d, *J* = 6.9 Hz, L-Ala-H-3), 1.61-1.73 (3H, m, D-Leu-H-3, 4), 1.45 (1H, m, HMDA-H-5a), 1.08-1.29 (8H, m, HMDA-H-6, 7, 8, 9), 0.97 (1H, m, HMDA-H-5b), 0.90 (3H, d, *J* = 5.9 Hz, D-Leu-H-5), 0.81-0.84 (6H, m, D-Leu-H-6, HMDA-H-10), 0.70 (3H, d, *J* = 6.9 Hz, HMDA-4-CH_3). ^{13}C-NMR (125 MHz, pyridine-*d*5): *δ* 171.8 (L-Phe-C-1), 171.6 (HMDA-C-1), 171.5 (L-Ala-C-1), 170.0 (D-Leu-C-1), 138.0 (L-Phe-C-4), 129.5 (L-Phe-C-5, 9), 128.5 (L-Phe-C-6, 8), 126.7 (L-Phe-C-7), 76.1 (HMDA-C-3), 57.5 (L-Phe-C-2), 53.0 (D-Leu-C-2), 50.1 (L-Ala-C-2), 41.6 (D-Leu-C-3), 36.6 (HMDA-C-2), 36.2 (L-Phe-C-3), 36.0 (HMDA-C-4), 31.7 (HMDA-C-8), 31.0 (HMDA-C-5), 29.5 (HMDA-C-7), 27.3 (HMDA-C-6), 25.0 (D-Leu-C-4), 22.6 (HMDA-C-9), 22.3 (D-Leu-C-5), 22.1 (D-Leu-C-6), 15.7 (L-Ala-C-3), 15.3 (HMDA-4-CH_3), 14.0 (HMDA-C-10).

Beauveriolide III: ^{1}H-NMR (500 MHz, pyridine-*d*5): *δ* 10.05 (1H, d, *J* = 7.0 Hz, L-Phe-2-NH), 9.95 (1H, d, *J* = 6.5 Hz, L-Ala-2-NH), 7.31 (1H, d, *J* = 9.4 Hz, D-allo-Ile-2-NH), 7.24 (5H, m, L-Phe-H-5, 6, 7, 8, 9), 5.23 (1H, m, HMOA-H-3), 4.83-4.90 (2H, m, L-Phe-H-2, D-allo-Ile-H-2), 4.19 (1H, m, L-Ala-H-2), 3.32 (1H, dd, *J* = 13.2, 8.9 Hz, L-Phe-H-3a), 3.14 (1H, dd, *J* = 13.2, 7.3 Hz, L-Phe-H-3b), 2.76 (1H, dd, *J* = 13.0, 3.6 Hz, HMOA-H-2a), 2.68 (1H, t, *J* = 12.0 Hz, HMOA-H-2b), 2.00 (1H, br s, HMOA-H-4), 1.81 (1H, m, D-allo-Ile-H-3), 1.70 (3H, d, *J* = 6.9 Hz, L-Ala-H-3), 1.45-1.47 (2H, m, HMOA-H-5a, D-allo-Ile-H-4a), 1.13-1.23 (5H, m, HMOA-H-6, 7, D-allo-Ile-H-4b), 0.96 (1H, m, HMOA-H-5b), 0.90 (3H, d, *J* = 6.6 Hz, D-allo-Ile-3-CH_3), 0.78-0.83 (6H, m, HMOA-H-8, D-allo-Ile-H-5), 0.66 (3H, d, *J* = 6.8 Hz, HMOA-4-CH_3). ^{13}C-NMR (125 MHz, pyridine-*d*5): *δ* 171.8 (L-Phe-C-1), 171.7 (HMOA-C-1), 171.6 (L-Ala-C-1), 169.7 (D-allo-Ile-C-1), 137.9 (L-Phe-C-4), 129.5 (L-Phe-C-5, 9), 128.5 (L-Phe-C-6, 8), 126.7 (L-Phe-C-7), 76.1 (HMOA-C-3), 59.7 (D-allo-Ile-C-2), 57.2 (L-Phe-C-2), 50.1 (L-Ala-C-2), 37.4 (D-allo-Ile-C-3), 36.5 (HMOA-C-2), 36.2 (L-Phe-C-3), 36.1 (HMOA-C-4),

30.5 (HMOA-C-5), 29.7 (HMOA-C-6), 26.1 (D-allo-Ile-C-4), 23.0 (HMOA-C-7), 15.6 (L-Ala-C-3), 15.2 (HMOA-4-CH_3), 14.8 (D-allo-Ile-3-CH_3), 14.0 (HMOA-C-8), 11.0 (D-allo-Ile-H-5).

Beauveriolide V: ^{1}H-NMR (500 MHz, pyridine-*d*5): *δ* 9.95 (1H, d, *J* = 7.5 Hz, L-Val-2-NH), 7.33 (1H, d, *J* = 9.4 Hz, D-allo-Ile-2-NH), 5.23 (1H, m, HMOA-H-3), 4.91 (1H, t, *J* = 10.0 Hz, D-allo-Ile-H-2), 4.25-4.32 (2H, m, L-Val-H-2, L-Ala-H-2), 2.88 (1H, dd, *J* = 13.4, 4.1 Hz, HMOA-H-2a), 2.77 (1H, dd, *J* = 13.0, 11.6 Hz, HMOA-H-2b), 2.39 (1H, br s, L-Val-H-3), 2.07 (1H, br s, HMOA-H-4), 1.85 (4H, m, L-Ala-H-3, D-allo-Ile-H-3), 1.48 (2H, m, HMOA-H-5a, D-allo-Ile-H-4a), 1.14-1.24 (5H, m, HMOA-H-6, 7, D-allo-Ile-H-4b), 0.94-1.00 (10H, m, HMOA-H-5b, D-Val-H-4, 5, D-allo-Ile-3-CH_3), 0.82-0.85 (6H, m, HMOA-H-8, D-allo-Ile-H-5), 0.70 (3H, d, *J* = 6.8 Hz, HMOA-4-CH_3). ^{13}C-NMR (125 MHz, pyridine-*d*5): *δ* 172.5 (L-Val-C-1), 172.0 (HMOA-C-1), 171.9 (L-Ala-C-1), 169.9 (D-allo-Ile-C-1), 76.5 (HMOA-C-3), 62.1 (L-Val-C-2), 59.9 (D-allo-Ile-C-2), 50.4 (L-Ala-C-2), 37.7 (D-allo-Ile-C-3), 36.7 (HMOA-C-2), 36.3 (HMOA-C-4), 30.7 (HMOA-C-5), 29.9 (HMOA-C-6), 28.5 (L-Val-C-3), 26.3 (D-allo-Ile-C-4), 23.3 (HMOA-C-7), 19.6 (L-Val-H-4, 5), 16.0 (L-Ala-C-3), 15.5 (HMOA-4-CH_3), 15.0 (D-allo-Ile-3-CH_3), 14.2 (HMOA-C-8), 11.2 (D-allo-Ile-H-5).

Beauveriolide VI: ^{1}H-NMR (500 MHz, pyridine-*d*5): *δ* 9.89 (1H, d, *J* = 7.5 Hz, L-Val-2-NH), 7.60 (1H, s, D-Leu-2-NH), 5.25 (2H, m, HMOA-H-3, D-Leu-H-2), 4.38 (1H, br s, L-Ala-H-2), 4.23 (1H, br s, L-Val-H-2), 2.89 (1H, dd, *J* = 13.5, 4.2 Hz, HMOA-H-2a), 2.80 (1H, dd, *J* = 13.4, 10.7 Hz, HMOA-H-2b), 2.41 (1H, br s, L-Val-H-3), 2.15 (1H, br s, HMOA-H-4), 1.81 (3H, d, *J* = 6.5 Hz, L-Ala-H-3), 1.65-1.74 (3H, m, D-Leu-H-3, 4), 1.48 (1H, m, HMOA-H-5a), 1.19 (4H, m, HMOA-H-6, 7), 0.97-1.00 (7H, m, HMOA-H-5b, D-Val-H-4, 5), 0.92 (3H, d, *J* = 5.8 Hz, D-Leu-H-5), 0.86 (3H, d, *J* = 5.8 Hz, D-Leu-H-6), 0.81 (3H, d, *J* = 6.9 Hz, HMOA-H-8), 0.72 (3H, d, *J* = 6.9 Hz, HMOA-4-CH_3). ^{13}C-NMR (125 MHz, pyridine-*d*5): *δ* 172.5 (L-Val-C-1), 171.8 (HMOA-C-1, L-Ala-C-1), 170.3 (D-Leu-C-1), 76.5 (HMOA-C-3), 62.1 (L-Val-C-2), 53.2 (D-Leu-C-2), 50.4 (L-Ala-C-2), 41.8 (D-Leu-C-3), 36.8 (HMOA-C-2), 36.2 (HMOA-C-4), 31.0 (HMOA-C-5), 29.7 (HMOA-C-6), 28.6 (L-Val-C-3), 25.2 (D-Leu-C-4), 23.2 (HMOA-C-7), 22.5 (D-Leu-C-5), 22.4 (D-Leu-C-6), 19.6 (L-Val-H-4, 5), 16.1 (L-Ala-C-3), 15.6 (HMOA-4-CH_3), 14.2 (HMOA-C-8).

Beauveriolide VII: ^{1}H-NMR (500 MHz, pyridine-*d*5): *δ* 10.07 (1H, d, *J* = 7.5 Hz, L-Phe-2-NH), 9.95 (1H, d, *J* = 7.1 Hz, L-Ala-2-NH), 7.38 (1H, d, *J* = 9.6 Hz, D-Val-2-NH), 7.25-7.27 (5H, m, L-Phe-H-5, 6, 7, 8, 9), 5.25 (1H, m, HMOA-H-3), 4.82-4.85 (2H, m, L-Phe-H-2, D-Val-H-2), 4.22 (1H, m, L-Ala-H-2), 3.35 (1H, dd, *J* = 13.3, 9.0 Hz, L-Phe-H-3a), 3.18 (1H, dd, *J* = 13.5, 7.2 Hz, L-Phe-H-3b), 2.79 (1H, dd, *J* = 13.2, 4.1 Hz, HMOA-H-2a), 2.71 (1H, dd, *J* = 13.1, 11.5 Hz, HMOA-H-2b), 2.03 (2H, m, HMOA-H-4, D-Val-H-3), 1.73 (3H, d, *J* = 6.9 Hz, L-Ala-H-3), 1.49 (1H, m, HMOA-H-5a), 1.18-1.26 (4H, m, HMOA-H-6, 7), 0.97 (1H, m, HMOA-H-5b), 0.96 (6H, m, D-Val-H-4, 5), 0.80 (3H, t, *J* = 7.0 Hz, HMOA-H-8), 0.69 (3H, d, *J* = 6.9 Hz, HMOA-4-CH_3). ^{13}C-NMR (125 MHz, pyridine-*d*5): *δ* 172.0 (HMOA-C-1, L-Phe-C-1), 171.9 (L-Ala-C-1), 169.8 (D-Val-C-1), 138.2 (L-Phe-C-4), 129.7 (L-Phe-C-5, 9), 128.8 (L-Phe-C-6, 8), 127.0 (L-Phe-C-7), 76.4 (HMOA-C-3), 61.1 (D-Val-C-2), 57.5 (L-Phe-C-2), 50.4 (L-Ala-C-2), 36.9 (HMOA-C-2), 36.4 (HMOA-C-4, L-Phe-C-3), 31.3 (D-Val-C-3), 30.8 (HMOA-C-5), 29.8 (HMOA-C-6), 23.1 (HMOA-C-7), 19.2 (D-Val-C-5), 19.0 (D-Val-C-4), 15.9 (L-Ala-C-3), 15.5 (HMOA-4-CH_3), 14.1 (HMOA-C-8).

Beauveriolide IX: ^{1}H-NMR (500 MHz, pyridine-*d*5): *δ* 10.30 (1H, d, *J* = 6.5 Hz, L-Phe-2-2-NH), 10.09 (1H, d, *J* = 7.0 Hz, L-Phe-1-2-NH), 7.34 (1H, d, *J* = 9.6 Hz, D-allo-Ile-2-NH), 7.26

(6H, m, L-Phe-1-H-6, 7, 8, L-Phe-2-H-6, 7, 8), 7.18 (2H, m, L-Phe-1-H-5, 9), 7.05 (2H, m, L-Phe-2-H-5, 9), 5.27 (1H, m, HMOA-H-3), 4.94 (2H, m, L-Phe-1-H-2, D-allo-Ile-H-2), 4.29 (1H, m, L-Phe-2-H-2), 3.94 (1H, dd, *J* = 13.9, 3.4 Hz, L-Phe-2-H-3a), 3.65 (1H, dd, *J* = 13.9, 10.9 Hz, L-Phe-2-H-3b), 3.34 (1H, dd, *J* = 13.5, 8.3 Hz, L-Phe-1-H-3a), 3.16 (1H, dd, *J* = 13.5, 7.4 Hz, L-Phe-1-H-3b), 2.82 (1H, dd, *J* = 13.2, 4.4 Hz, HMOA-H-2a), 2.72 (1H, dd, *J* = 13.1, 11.7 Hz, HMOA-H-2b), 2.11 (1H, m, HMOA-H-4), 1.84 (1H, m, D-allo-Ile-H-3), 1.50 (2H, m, HMOA-H-5a, D-allo-Ile-H-4a), 1.18-1.27 (4H, m, HMOA-H-6, 7), 1.00 (2H, m, HMOA-H-5b, D-allo-Ile-H-4b), 0.95 (3H, d, *J* = 6.7 Hz, D-allo-Ile-3-CH_3), 0.82-0.87 (6H, m, HMOA-H-8, D-allo-Ile-H-5), 0.70 (3H, d, *J* = 6.9 Hz, HMOA-4-CH_3).

Beauveriolide X: ^{1}H-NMR (500 MHz, pyridine-*d*5): *δ* 10.18 (1H, d, *J* = 7.3 Hz, L-Phe-2-2-NH), 10.06 (1H, d, *J* = 7.2 Hz, L-Phe-1-2-NH), 7.60 (1H, s, D-Leu-2-NH), 7.26 (6H, m, L-Phe-1-H-6, 7, 8, L-Phe-2-H-6, 7, 8), 7.19 (2H, m, L-Phe-1-H-5, 9), 7.08 (2H, m, L-Phe-2-H-5, 9), 5.28 (2H, m, HMOA-H-3, D-Leu-H-2), 4.98 (1H, s, L-Phe-1-H-2), 4.30 (1H, m, L-Phe-2-H-2), 3.90 (1H, dd, *J* = 13.8, 3.8 Hz, L-Phe-2-H-3a), 3.63 (1H, dd, *J* = 13.8, 10.8 Hz, L-Phe-2-H-3b), 3.36 (1H, dd, *J* = 13.3, 8.5 Hz, L-Phe-1-H-3a), 3.20 (1H, dd, *J* = 13.4, 7.5 Hz, L-Phe-1-H-3b), 2.84 (1H, dd, *J* = 13.3, 4.3 Hz, HMOA-H-2a), 2.74 (1H, dd, *J* = 13.3, 10.9 Hz, HMOA-H-2b), 2.08 (1H, m, HMOA-H-4), 1.67-1.74 (3H, m, D-Leu-H-3, 4), 1.47 (1H, m, HMOA-H-5a), 1.18-1.25 (4H, m, HMOA-H-6, 7), 0.99 (1H, m, HMOA-H-5b), 0.93 (3H, d, *J* = 6.1 Hz, D-Leu-H-5), 0.86 (3H, d, *J* = 6.2 Hz, D-Leu-H-6), 0.81 (3H, d, *J* = 7.0 Hz, HMOA-H-8), 0.72 (3H, d, *J* = 6.9 Hz, HMOA-4-CH_3). ^{13}C-NMR (125 MHz, pyridine-*d*5): *δ* 172.1 (L-Phe-1-C-1), 171.6 (HMOA-C-1), 170.8 (L-Phe-2-C-1), 170.3 (D-Leu-C-1), 140.7 (L-Phe-2-C-4), 138.1 (L-Phe-1-C-4), 130.0 (L-Phe-2-C-5, 9), 129.8 (L-Phe-1-C-5, 9), 128.9 (L-Phe-1-C-6, 8, L-Phe-2-C-6, 8), 127.1 (L-Phe-1-C-7), 126.4 (L-Phe-2-C-7), 76.2 (HMOA-C-3), 57.8 (L-Phe-2-C-2), 56.6 (L-Phe-1-C-2), 53.2 (D-Leu-C-2), 41.9 (D-Leu-C-3), 37.2 (HMOA-C-2), 36.6 (L-Phe-1-C-3), 36.4 (HMOA-C-4), 35.4 (L-Phe-2-C-3), 31.0 (HMOA-C-5), 29.7 (HMOA-C-6), 25.2 (D-Leu-C-4), 23.2 (HMOA-C-7), 22.5 (D-Leu-C-5), 22.4 (D-Leu-C-6), 15.5 (HMOA-4-CH_3), 14.2 (HMOA-C-8). Beauveriolide X is a new homolog of beauveriolides, compared with beauveriolide IX, it has D-Leu instead of D-allo-Ile.

Beauverolide Ba: ^{1}H-NMR (500 MHz, pyridine-*d*5): *δ* 9.94 (1H, d, *J* = 7.7 Hz, L-Val-2-NH), 7.45 (1H, d, *J* = 9.5 Hz, D-allo-Ile-2-NH), 7.42 (2H, d, *J* = 7.0 Hz, L-Phe-H-5, 9), 7.34 (2H, t, *J* = 7.0 Hz, L-Phe-H-6, 8), 7.28 (1H, t, *J* = 7.3 Hz, L-Phe-H-7), 5.26 (1H, m, HMDA-H-3), 4.97 (1H, t, *J* = 9.8 Hz, D-allo-Ile-H-2), 4.55 (1H, br s, L-Phe-H-2), 4.24 (1H, br s, L-Val-H-2), 4.01 (1H, br d, L-Phe-H-3a), 3.75 (1H, br s, L-Phe-H-3b), 2.92 (1H, dd, *J* = 13.4, 4.2 Hz, HMDA-H-2a), 2.81 (1H, dd, *J* = 13.2, 11.5 Hz, HMDA-H-2b), 2.35 (1H, br s, L-Val-H-3), 2.11 (1H, m, HMDA-H-4), 1.89 (1H, m, D-allo-Ile-H-3), 1.50 (2H, m, HMDA-H-5a, D-allo-Ile-H-4a), 1.16-1.21 (8H, m, HMDA-H-6a, 7, 8, 9, D-allo-Ile-H-4b), 1.04-1.09 (2H, m, HMDA-H-5b, 6b), 0.97 (3H, d, *J* = 6.7 Hz, D-allo-Ile-3-CH_3), 0.95 (3H, d, *J* = 6.6 Hz, L-Val-H-4), 0.83-0.88 (9H, m, HMDA-H-8, L-Val-H-5, D-allo-Ile-H-5), 0.74 (3H, d, *J* = 6.9 Hz, HMDA-4-CH_3). ^{13}C-NMR (125 MHz, pyridine-*d*5): *δ* 172.5 (L-Val-C-1), 171.5 (HMDA-C-1), 171.1 (L-Phe-C-1), 169.8 (D-allo-Ile-C-1), 140.6 (L-Phe-C-4), 130.0 (L-Phe-C-5, 9), 128.7 (L-Phe-C-6, 8), 126.4 (L-Phe-C-7), 76.4 (HMDA-C-3), 62.0 (L-Val-C-2), 59.8 (D-allo-Ile-C-2), 57.4 (L-Phe-C-2), 37.7 (D-allo-Ile-C-3), 36.8 (HMDA-C-2), 36.2 (HMDA-C-4), 35.6 (L-Phe-C-3), 31.9 (HMDA-C-8), 31.0 (HMDA-C-5), 29.8 (HMDA-C-7), 28.3 (L-Val-C-3), 27.5 (HMDA-C-6), 26.2 (D-allo-Ile-C-4), 22.8 (HMDA-C-9), 19.7 (L-Val-C-5), 19.4 (L-Val-C-4), 15.4 (HMDA-4-CH_3), 15.0

(D-allo-Ile-3-CH_3), 14.1 (HMDA-C-10), 11.2 (D-allo-Ile-C-5).

Beauverolide Ca: ^{1}H-NMR (500 MHz, pyridine-*d*5): *δ* 10.31 (1H, d, *J* = 6.7 Hz, L-Phe-2-2-NH), 10.10 (1H, d, *J* = 7.1 Hz, L-Phe-1-2-NH), 7.34 (1H, d, *J* = 9.7 Hz, D-allo-Ile-2-NH), 7.24 (6H, m, L-Phe-1-H-6, 7, 8, L-Phe-2-H-6, 7, 8), 7.15 (2H, m, L-Phe-1-H-5, 9), 7.01 (2H, m, L-Phe-2-H-5, 9), 5.26 (1H, m, HMDA-H-3), 4.95-5.01 (2H, m, L-Phe-1-H-2, D-allo-Ile-H-2), 4.26 (1H, m, L-Phe-2-H-2), 3.91 (1H, dd, *J* = 13.7, 3.2 Hz, L-Phe-2-H-3a), 3.64 (1H, dd, *J* = 13.3, 11.2 Hz, L-Phe-2-H-3b), 3.32 (1H, dd, *J* = 13.4, 8.2 Hz, L-Phe-1-H-3a), 3.13 (1H, dd, *J* = 13.4, 7.1 Hz, L-Phe-1-H-3b), 2.82 (1H, dd, *J* = 13.0, 4.3 Hz, HMDA-H-2a), 2.72 (1H, q, *J* = 12.1 Hz, HMDA-H-2b), 2.00 (1H, m, HMDA-H-4), 1.83 (1H, m, D-allo-Ile-H-3), 1.47 (2H, m, HMDA-H-5a, D-allo-Ile-H-4a), 1.15-1.20 (9H, m, HMDA-H-6, 7, 8, 9, D-allo-Ile-H-4b), 0.98 (1H, m, HMDA-H-5b), 0.93 (3H, d, *J* = 6.7 Hz, D-allo-Ile-3-CH_3), 0.81-0.84 (6H, m, HMDA-H-8, D-allo-Ile-H-5), 0.69 (3H, d, *J* = 7.0 Hz, HMDA-4-CH_3). ^{13}C-NMR (125 MHz, pyridine-*d*5): *δ* 171.6 (L-Phe-1-C-1), 171.4 (HMDA-C-1), 170.8 (L-Phe-2-C-1), 169.6 (D-allo-Ile-C-1), 140.6 (L-Phe-2-C-4), 137.8 (L-Phe-1-C-4), 129.8 (L-Phe-2-C-5, 9), 129.5 (L-Phe-1-C-5, 9), 128.6 (L-Phe-1-C-6, 8), 128.5 (L-Phe-2-C-6, 8), 126.8 (L-Phe-1-C-7), 126.1 (L-Phe-2-C-7), 76.1 (HMDA-C-3), 59.7 (D-allo-Ile-C-2), 57.6 (L-Phe-2-C-2), 56.0 (L-Phe-1-C-2), 37.7 (D-allo-Ile-C-3), 36.8 (HMDA-C-2), 36.4 (L-Phe-1-C-3), 36.2 (HMDA-C-4), 34.9 (L-Phe-2-C-3), 31.8 (HMDA-C-8), 30.8 (HMDA-C-5), 29.6 (HMDA-C-7), 27.4 (HMDA-C-6), 26.1 (D-allo-Ile-C-4), 22.7 (HMDA-C-9), 15.2 (HMDA-4-CH_3), 14.8 (D-allo-Ile-3-CH_3), 14.0 (HMDA-C-10), 11.0 (D-allo-Ile-C-5).

Beauverolide Ka: ^{1}H-NMR (500 MHz, pyridine-*d*5): *δ* 11.92 (1H, s, L-Trp-5-NH), 10.43 (1H, d, *J* = 6.9 Hz, L-Phe-2-NH), 10.07 (1H, d, *J* = 7.1 Hz, L-Trp-2-NH), 7.60 (1H, d, *J* = 8.2 Hz, L-Trp-H-7), 7.56 (1H, s, L-Trp-H-10), 7.38 (1H, d, *J* = 9.7 Hz, D-allo-Ile-2-NH), 7.29 (1H, s, L-Trp-H-5), 7.23 (1H, t, *J* = 7.6 Hz, L-Trp-H-8), 7.18 (3H, m, L-Phe-H-6, 7, 8), 6.97-7.00 (3H, m, L-Trp-H-9, L-Phe-H-5, 9), 5.31 (1H, dt, *J* = 11.7, 4.0 Hz, HMDA-H-3), 5.20 (1H, q, *J* = 7.7 Hz, L-Trp-H-2), 4.98 (1H, t, *J* = 9.9 Hz, D-allo-Ile-H-2), 4.21 (1H, m, L-Phe-H-2), 3.90 (1H, dd, *J* = 13.9, 3.1 Hz, L-Phe-H-3a), 3.59-3.67 (2H, m, L-Trp-H-3a, L-Phe-H-3b), 3.42 (1H, dd, *J* = 14.2, 7.2 Hz, L-Trp-H-3b), 2.85 (1H, dd, *J* = 13.2, 4.2 Hz, HMDA-H-2a), 2.72 (1H, t, *J* = 12.2 Hz, HMDA-H-2b), 2.03 (1H, m, HMDA-H-4), 1.86 (1H, m, D-allo-Ile-H-3), 1.50 (2H, m, HMDA-H-5a, D-allo-Ile-H-4a), 1.16-1.22 (8H, m, HMDA-H-6a, 7, 8, 9, D-allo-Ile-H-4b), 1.02-1.10 (2H, m, HMDA-H-5b, 6b), 0.95 (3H, d, *J* = 6.6 Hz, D-allo-Ile-3-CH_3), 0.85-0.88 (6H, m, HMDA-H-8, D-allo-Ile-H-5), 0.71 (3H, d, *J* = 6.9 Hz, HMDA-4-CH_3). ^{13}C-NMR (125 MHz, pyridine-*d*5): *δ* 172.3 (L-Trp-C-1), 171.4 (HMDA-C-1), 171.0 (L-Phe-C-1), 169.7 (D-allo-Ile-C-1), 140.7 (L-Phe-C-4), 137.4 (L-Trp-C-6), 129.8 (L-Phe-C-5, 9), 128.5 (L-Phe-C-6, 8), 128.1 (L-Trp-C-11), 126.1 (L-Phe-C-7), 124.4 (L-Trp-C-5), 121.6 (L-Trp-C-8), 119.0 (L-Trp-C-9), 118.8 (L-Trp-C-10), 111.8 (L-Trp-C-7), 110.7 (L-Trp-C-4), 76.1 (HMDA-C-3), 59.7 (D-allo-Ile-C-2), 57.7 (L-Phe-C-2), 55.5 (L-Trp-C-2), 37.7 (D-allo-Ile-C-3), 36.8 (HMDA-C-2), 36.2 (HMDA-C-4), 34.9 (L-Phe-C-3), 31.8 (HMDA-C-8), 30.8 (HMDA-C-5), 29.6 (HMDA-C-7), 27.5 (HMDA-C-6), 26.7 (L-Trp-C-3), 26.1 (D-allo-Ile-C-4), 22.8 (HMDA-C-9), 15.2 (HMDA-4-CH_3), 14.8 (D-allo-Ile-3-CH_3), 14.0 (HMDA-C-10), 11.2 (D-allo-Ile-C-5).

Beauverolide La: ^{1}H-NMR (500 MHz, pyridine-*d*5): *δ* 10.07 (1H, d, *J* = 7.1 Hz, L-Phe-2-NH), 9.97 (1H, d, *J* = 7.1 Hz, L-Ala-2-NH), 7.36 (1H, d, *J* = 9.4 Hz, D-allo-Ile-2-NH), 7.22-7.24 (5H, m, L-Phe-H-5, 6, 7, 8, 9), 5.26 (1H, m, HMDA-H-3), 4.89 (1H, m, D-allo-Ile-H-2), 4.83 (1H, q, *J* = 7.8 Hz, L-Phe-H-2), 4.20 (1H, m, L-Ala-H-2), 3.32 (1H, dd, *J* = 13.4, 8.6 Hz, L-Phe-H-

3a), 3.14 (1H, dd, J = 13.3, 7.5 Hz, L-Phe-H-3b), 2.78 (1H, dd, J = 13.4, 4.2 Hz, HMDA-H-2a), 2.70 (1H, dd, J = 13.4, 10.9 Hz, HMDA-H-2b), 2.09 (1H, m, HMDA-H-4), 1.83 (1H, m, D-allo-Ile-H-3), 1.70 (3H, d, J = 6.9 Hz, L-Ala-H-3), 1.46-1.48 (2H, m, HMDA-H-5a, D-allo-Ile-H-4a), 1.08-1.29 (9H, m, HMDA-H-6, 7, 8, 9, D-allo-Ile-H-4b), 0.98 (1H, m, HMDA-H-5b), 0.91 (3H, d, J = 6.6 Hz, D-allo-Ile-3-CH_3), 0.82-0.85 (6H, m, HMDA-H-10, D-allo-Ile-H-5), 0.69 (3H, d, J = 6.9 Hz, HMDA-4-CH_3). ^{13}C-NMR (125 MHz, pyridine-*d*5): δ 171.8 (HMDA-C-1, L-Phe-C-1), 171.7 (L-Ala-C-1), 169.7 (D-allo-Ile-C-1), 137.9 (L-Phe-C-4), 129.5 (L-Phe-C-5, 9), 128.5 (L-Phe-C-6, 8), 126.7 (L-Phe-C-7), 76.1 (HMDA-C-3), 59.7 (D-allo-Ile-C-2), 57.1 (L-Phe-C-2), 50.2 (L-Ala-C-2), 37.4 (D-allo-Ile-C-3), 36.5 (HMDA-C-2), 36.2 (L-Phe-C-3), 36.1 (HMDA-C-4), 31.7 (HMDA-C-8), 30.8 (HMDA-C-5), 29.6 (HMDA-C-7), 27.4 (HMDA-C-6), 26.1 (D-allo-Ile-C-4), 22.6 (HMDA-C-9), 15.6 (L-Ala-C-3), 15.2 (HMDA-4-CH_3), 14.8 (D-allo-Ile-3-CH_3), 14.0 (HMDA-C-10), 11.0 (D-allo-Ile-H-5).
