# Supplementary figures and images for: Production of Diverse Beauveriolide Analogs in Closely Related Fungi: a Rare Case of Fungal Chemodiversity
Source: mSphere. 2020 Sep 2;5(5):e00667-20. doi: 10.1128/mSphere.00667-20 (PMC7471007; doi:10.1128/mSphere.00667-20)

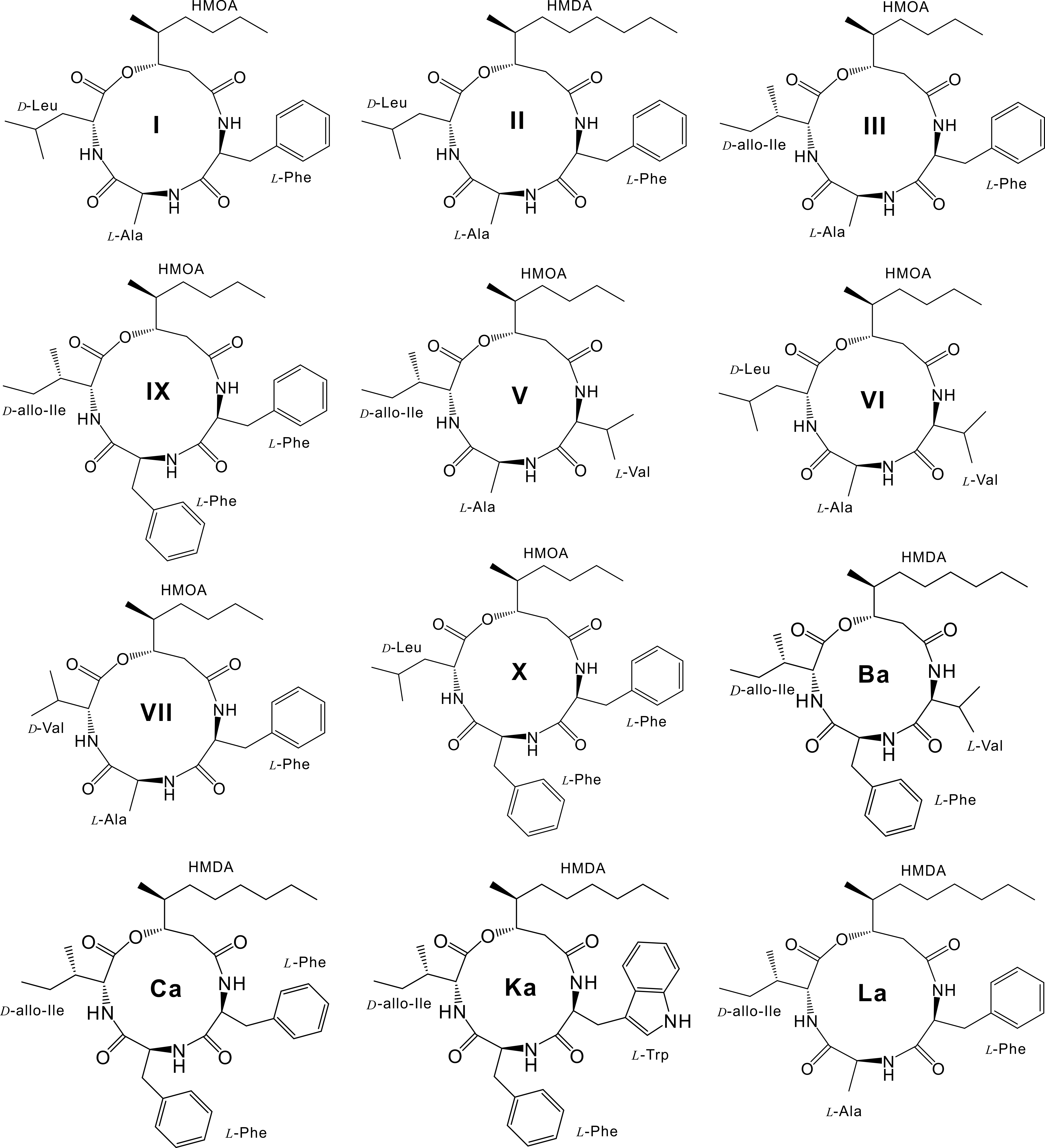

Supplement: FIG S1 [file mSphere.00667-20-sf001.tif]

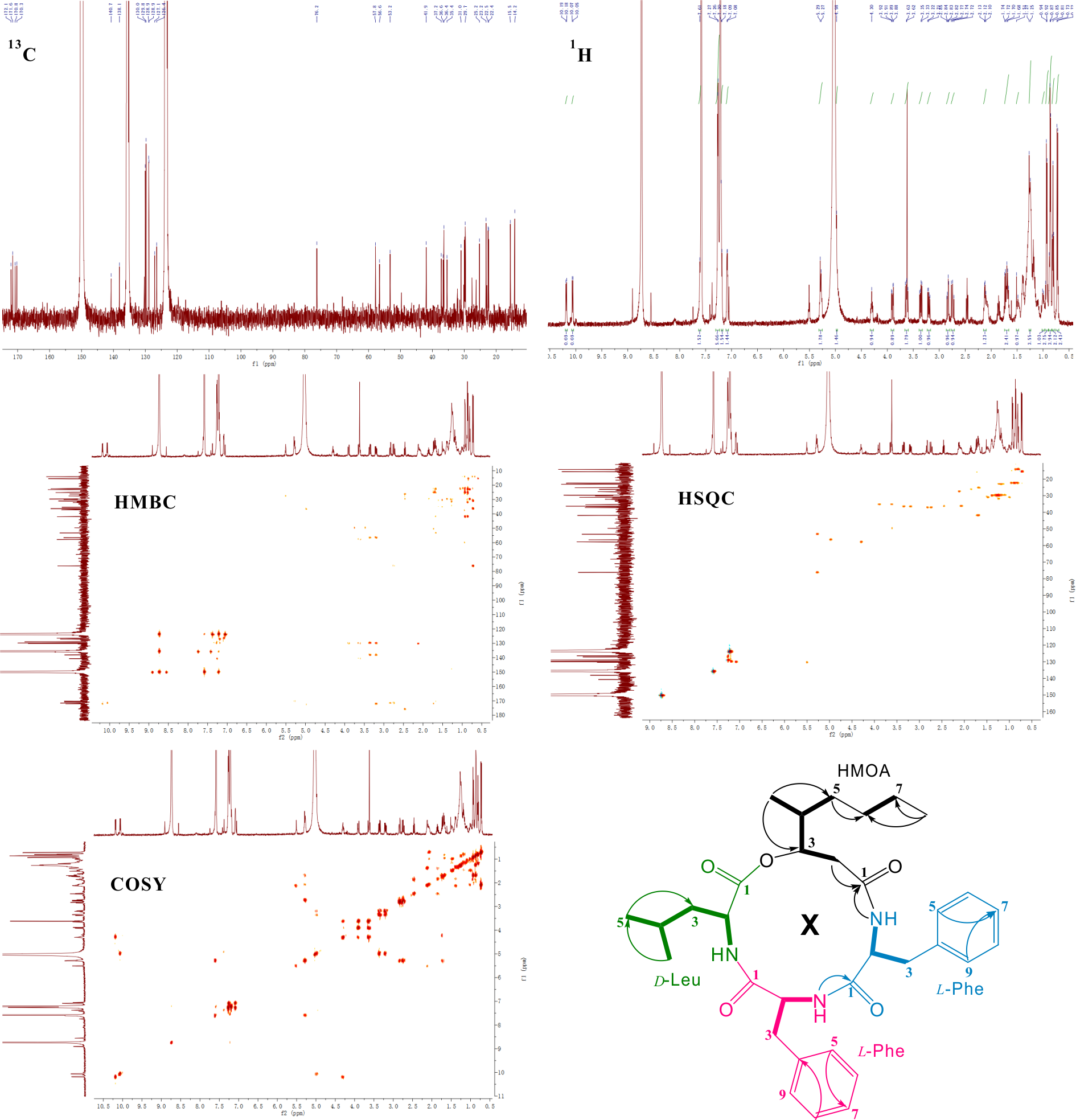

Supplement: FIG S2 [file mSphere.00667-20-sf002.tif]

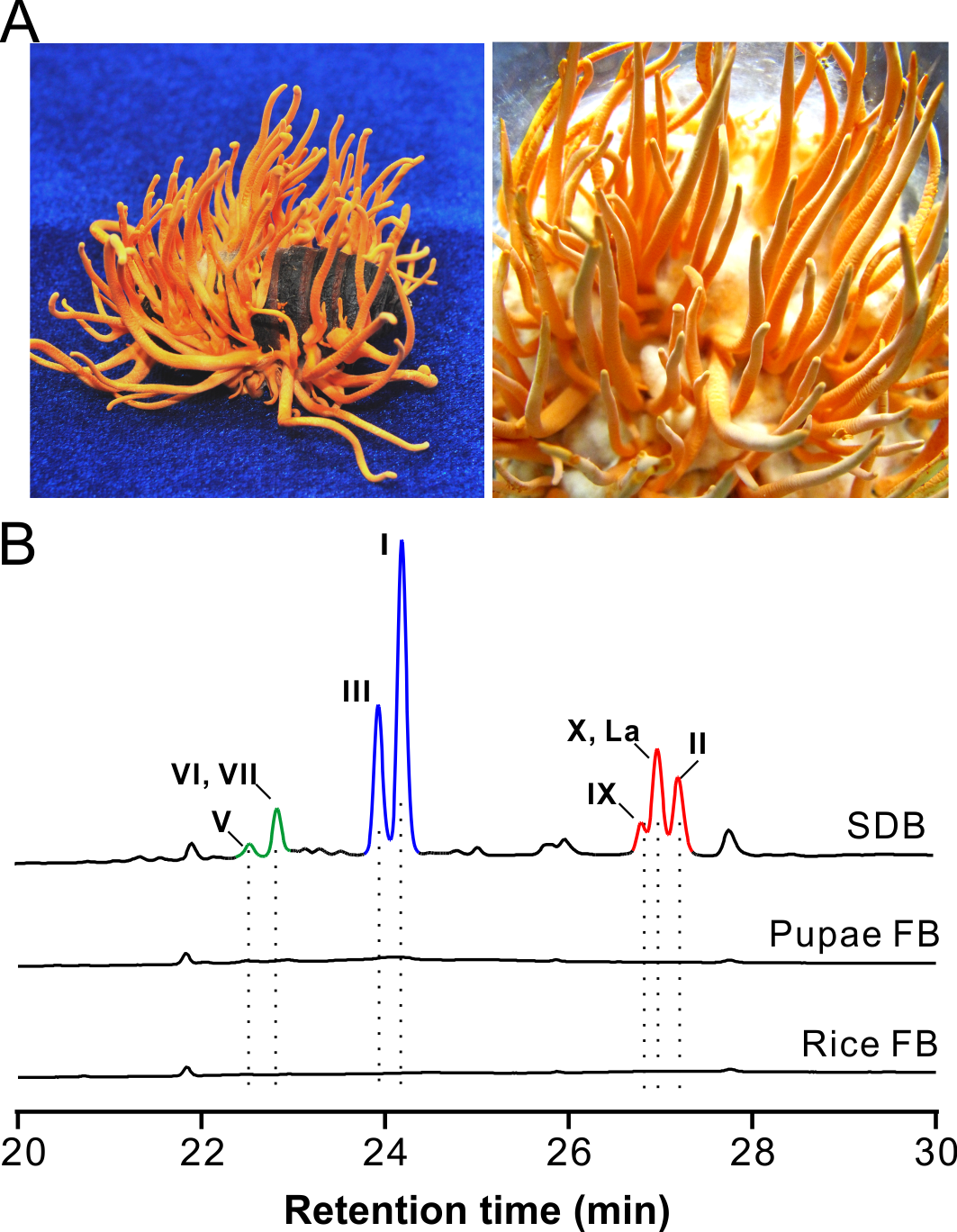

Supplement: FIG S3 [file mSphere.00667-20-sf003.tif]

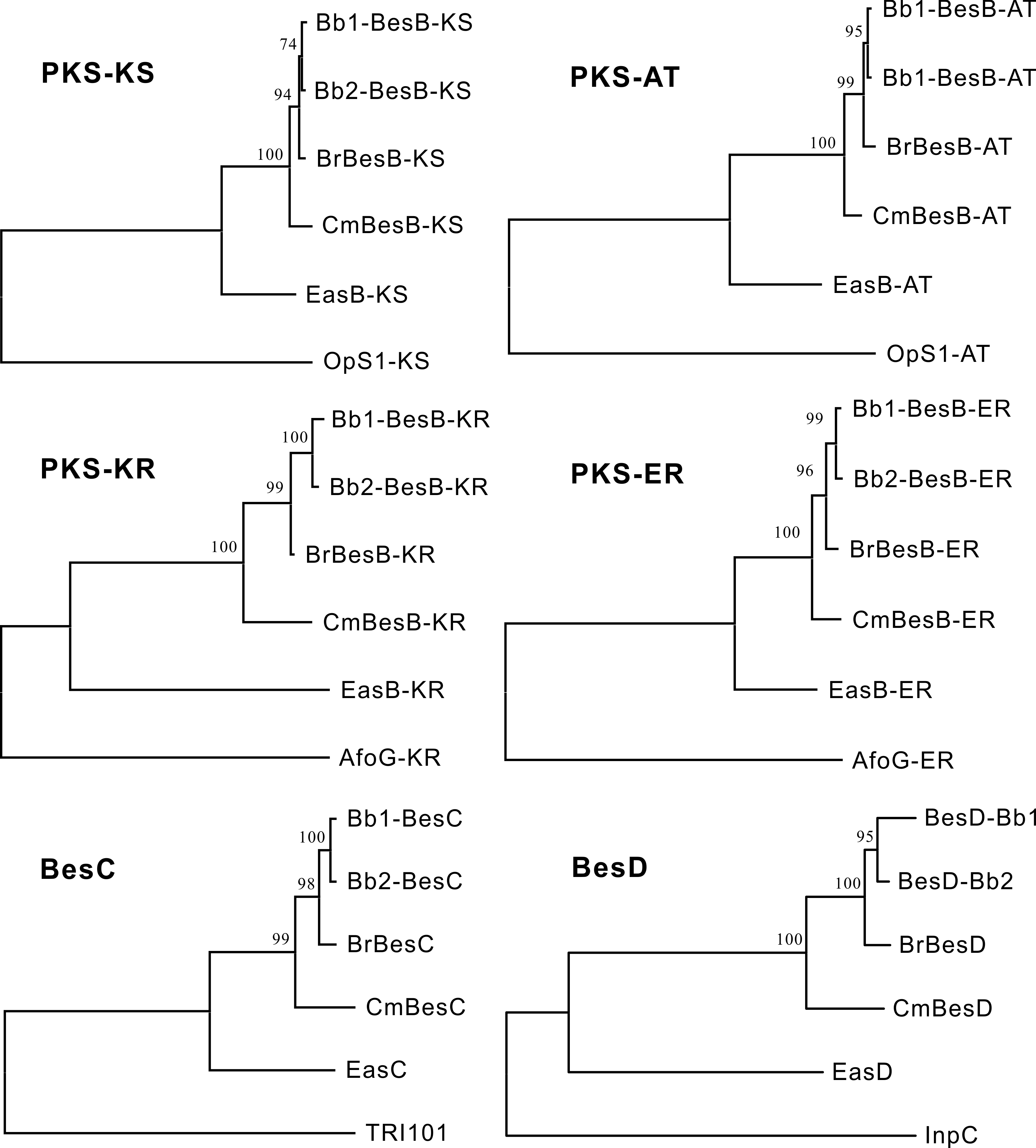

Supplement: FIG S4 [file mSphere.00667-20-sf004.tif]
