# Supplementary material for: Production of Diverse Beauveriolide Analogs in Closely Related Fungi: a Rare Case of Fungal Chemodiversity
Source: mSphere. 2020 Sep 2;5(5):e00667-20. doi: 10.1128/mSphere.00667-20 (PMC7471007; doi:10.1128/mSphere.00667-20)
Supplement: TABLE S2 [file mSphere.00667-20-st002.pdf]

Table S2 Putative compositions of the compounds obtained from the mutant Δ*CmbesB* fed with 3-HDA and 3-HOA.

**HPLC peaks***	**Putative compounds**	**n**	**R**	***L*-X**	***L*-Y**	***D*-Z**	**MW****
Peaks 1	Dm-La	5	H	Phe	Ala	allo-Ile	501
	Dm-L (II)	5	H	Phe	Ala	Leu	501
Peaks 2	Dm-V	3	H	Val	Ala	allo-Ile	425
	Dm-VI	3	H	Val	Ala	Leu	425
	Dm-VII	3	H	Phe	Ala	Val	459
Peaks 3	Dm-III	3	H	Phe	Ala	allo-Ile	473
	Dm-I	3	H	Phe	Ala	Leu	473
Peaks 4	Dm-IX	3	H	Phe	Phe	allo-Ile	549
	Dm-X	3	H	Phe	Phe	Leu	549

*, Peaks are as shown in Fig. 3B. **, MW, molecular weight. Dm, demethyl.
