# Supplementary material for: Production of Diverse Beauveriolide Analogs in Closely Related Fungi: a Rare Case of Fungal Chemodiversity
Source: mSphere. 2020 Sep 2;5(5):e00667-20. doi: 10.1128/mSphere.00667-20 (PMC7471007; doi:10.1128/mSphere.00667-20)
Supplement: TABLE S3 [file mSphere.00667-20-st003.pdf]

Table S3 PCR Primers used in this study.

Genes	Name	Primer sequence	RE*	Note
besA BBA_08222	U1	CGGGATCCCGACCTGCAACCTAGTTTTT	BamH I	Gene deletion
	U2	CGGGATCCACTGACATGTCCCCTGAGTT		
	L1	GGACTAGTCGATGTCTGCTTTTCATACG	Spe I	
	L2	GGACTAGTAGTGCATCATCAATCACGAC		
	F	CTCCCTTTCTATGGCGAATA		PCR verification
	R	TCTGAAAGGTCATGATGGTG		
besB BBA_08219	U1	CGGGATCCAGATGGAAGTGGCTTTCTTG	BamH I	Gene deletion
	U2	CGGGATCCTGCTTCAGAAACCTGACAGA		
	L1	GGACTAGTAGTCGACAACTCATCCCAAT	Spe I	
	L2	GGACTAGTCGCCCAAAAAGTAACAAACT		
	F	GCGTCTACAACAACATGAGC		PCR verification
	R	TGTTGTGCATTTTCAGATCC		
besC BBA_08220	U1	CGGAATTCGACTCGTTGCTTTGTCTGTC	EcoR I	Gene deletion
	U2	CGGGATCCCGAGCACAAGATTGGCATAG	BamH I	
	L1	GGACTAGTAGTGACGCTGTGCATTTTAC	Spe I	
	L2	GGACTAGTGCAACTGTACTTCGAAACCC		
	F	CTTTTCTCGGGCTCATTGTG		PCR verification
	R	CTGTCACGACTAGATCAGGG		
besD BBA_08221	U1	GGACTAGTTGCGAGGACTAAGAACAGAC	Spe I	Gene deletion
	U2	GGACTAGTATCAAGGTCAAGGTGAGTGG		
	L1	CGGGATCCGTGGAACTATGGGATGCAGA	BamH I	
	L2	CGGAATTCACACTAGCTGACACCTCATG	EcoR I	
	F	TAGAGTCATCGTTGGTCAGC		PCR verification
	R	TGGATGAAAATGCAACGACC		
BrbesA BBO_00138	U1	GCTTGATATCGAATTGTTGGCTTGCTCGGTAAGAG	EcoR I	Fusion PCR for gene deletion
	U2	CGGGCTGCAGGAATTGTGGAACGATACTCGGCACT		
	L1	GATCTGATGAACTAGTGGGAGATCCATCAGGTAGG	Spe I	
	L2	CCGCTCTAGAACTAGCTACATATCCGCTGCTGCAA		
	F	CTCTCAACGAAAAGGGTTCG		PCR verification
	R	ACACGCTTCTGCACCAACTT		
BrbesB BBO_00141	U1	GCTTGATATCGAATTATTCGTAAAGCCCCCTTTGT	EcoR I	Fusion PCR for gene deletion
	U2	CGGGCTGCAGGAATTGCAGTCTATATGGGGCGAGA		
	L1	GATCTGATGAACTAGGAAGCAAAGGTGAGCCACTC	Spe I	
	L2	CCGCTCTAGAACTAGCTGGCTGCACTGATGAAAAA		
	F	GGTGTACTTCAGCCGTATCCA		PCR verification
	R	CATTAGGCGAGCCCTATGAG		
CmbesA CCM_01285	U1	GCTTGATATCGAATTGAATCCGACTCCTTGGTTGA	EcoR I	Fusion PCR for gene deletion
	U2	CGGGCTGCAGGAATTCTAGGGCGACGAAAGACTTG		
	L1	GATCTGATGAACTAGCACGACAGGTAGAGCAACGA	Spe I	
	L2	CCGCTCTAGAACTAGCTTTTCGAAACACGTGCAGA		
	F	ACGGATGAGAACGAGCAAGT		PCR verification
	R	TGAGCGTCTCAAAATCGTTG		
CmbesB CCM_01282	U1	GCTTGATATCGAATTGCTCCGCTGATGAAAAAGAC	EcoR I	Fusion PCR for gene deletion
	U2	CGGGCTGCAGGAATTCTTTCCAGCTCCAAAACTGC		
	L1	GATCTGATGAACTAGCGCACAGTTTGAGTTGGAAA	Spe I	
	L2	CCGCTCTAGAACTAGGGGGAGAATACTTCCGGGTA		
	F	CTAGAAGAAGGCGGTGTTGG		PCR verification
	R	GGATATTGATCAGCCGCACT		
CmbesD CCM_01284	Dfus-F	TTTTAATCAATAACAATGATCTTTTCATCGCCAGC		Fusion PCR for gene complementation of Δ*besD*
	Dfus-R	CACGTCGACGGATCCTCAATTCGATGCCGCTTTCT		

*RE, restriction enzyme.
